# Pleiotropy robust methods for multivariable Mendelian randomization

**DOI:** 10.1002/sim.9156

**Published:** 2021-08-02

**Authors:** Andrew J. Grant, Stephen Burgess

**Affiliations:** 1 MRC Biostatistics Unit, University of Cambridge, Cambridge, UK; 2 Cardiovascular Epidemiology Unit, University of Cambridge, Cambridge, UK

**Keywords:** Mendelian randomization, multivariable, pleiotropy, robust estimation

## Abstract

Mendelian randomization is a powerful tool for inferring the presence, or otherwise, of causal effects from observational data. However, the nature of genetic variants is such that pleiotropy remains a barrier to valid causal effect estimation. There are many options in the literature for pleiotropy robust methods when studying the effects of a single risk factor on an outcome. However, there are few pleiotropy robust methods in the multivariable setting, that is, when there are multiple risk factors of interest. In this article we introduce three methods which build on common approaches in the univariable setting: MVMR-Robust; MVMR-Median; and MVMR-Lasso. We discuss the properties of each of these methods and examine their performance in comparison to existing approaches in a simulation study. MVMR-Robust is shown to outperform existing outlier robust approaches when there are low levels of pleiotropy. MVMR-Lasso provides the best estimation in terms of mean squared error for moderate to high levels of pleiotropy, and can provide valid inference in a three sample setting. MVMR-Median performs well in terms of estimation across all scenarios considered, and provides valid inference up to a moderate level of pleiotropy. We demonstrate the methods in an applied example looking at the effects of intelligence, education and household income on the risk of Alzheimer’s disease.

## 1 Introduction

Mendelian randomization is a technique for estimating the causal effect of a risk factor on an outcome using observational data.^1^ It uses genetic variants as instrumental variables and can provide valid causal effect estimation in the presence of unmeasured confounding. Three assumptions are required in order that a genetic variant is a valid instrument: it must be associated with the risk factor of interest; it must not be associated with any confounder of the risk factor-outcome relationship; and it must be independent of the outcome conditional on the risk factor and confounders.^2^

Genetic variants are good candidates for instrumental variables: they are naturally independent of many environmental factors which are common sources of confounding, and mitigate the potential for reverse causation. Furthermore, methods for Mendelian randomization have been developed which allow for combining many instruments in a single analysis, and which can also be used when only summary statistics of the associations between the genetic variants and traits are available.^3^ These features allow practitioners to harness publicly available summary data from genome wide association studies (GWAS). Two-sample approaches, where the genetic variant-risk factor and genetic variant-outcome associations are estimated in different samples, open up vast combinations of risk factor-outcome relationships to be studied.^4^ The major limitation in Mendelian randomization analyses is therefore the potential presence of pleiotropy, which is when genetic variants associate with traits other than the risk factor of interest. If any such trait provides an alternative causal pathway to the outcome not via the risk factor, then the corresponding genetic variants are invalid instruments and causal effect estimates may be biased.

Multivariable Mendelian randomization fits multiple risk factors in a single model.^5^ One motivation for its use is to account for pleiotropy in a univariable analysis via a set of measured covariates. It can be an important sensitivity analysis if there are known biological pathways linking the genetic variants and the outcome. If any such biological pathways bypass the risk factor, the genetic variants will not be independent of the outcome conditional on the risk factor and outcome, and the instrumental variables assumptions are violated. Another motivation is if there are a number of correlated traits with shared genetic predictors which are all hypothesized to have potential causal effects on the outcome. A multivariable model can distinguish between the direct effects of the risk factors on the outcome and the total effects inclusive of mediators.^6^ A genetic variant is a valid instrument for multivariable Mendelian randomization if: it is associated with at least one risk factor; it is independent of any confounder of each risk factor-outcome relationship; and it is independent of the outcome conditional on all risk factors and confounders. Causal pathways from a genetic variant to the outcome that do not pass via one or more of the risk factors are referred to as unmeasured pleiotropy (in contrast to measured pleiotropy, where such pathways are entirely accounted for via the set of risk factors). For the purposes of this article, we use the word pleiotropy to mean unmeasured pleiotropy.

There are a number methods in the literature for univariable Mendelian randomization (ie, when there is a single risk factor) which are robust to pleiotropy.^7^ Each method provides valid estimation of the causal effect under different sets of assumptions. Although these assumptions are, generally, untestable, an applied analysis will typically employ a range of methods. Consistency of results across various methods which rely on different assumptions gives strength of evidence to the findings.^8^ There are, however, few methods for pleiotropy robust multivariable Mendelian randomization.^9,10^ Valid estimation of causal effects, therefore, typically relies on the assumption that all causal pathways between the genetic variants and the outcome are accounted for via the measured risk factors.

In this article we propose a number of novel approaches to multivariable Mendelian randomization which provide robustness to different forms of pleiotropy. The methods are developed for use with summary level data, and so access to individual level data is not required. We examine the performance of the methods under various pleiotropic settings in a simulation study. We then demonstrate the methods in an applied analysis looking at the effects of intelligence, years of education and household income on the risk of Alzheimer’s disease.

## 2 Modeling Assumptions

### 2.1 Data generating model

We assume the following model, which is similar to a multivariable version of the models set out by Bowden et al^11^ and Kang et al^12^ in the single risk factor case. For individual *i*, let *Y_i_* be the outcome, *X_i_*1, …,*X_iK_* be *K* risk factors, *G*_*i*1_, …,*G_ip_* be *p* genetic variants and *U_i_* represent confounders of the risk factor-outcome relationships. The data generating model is:

(1) $$X_{ik}=\beta_{X0k}+\sum_{j=1}^{p}\beta_{Xjk}G_{ij}+\gamma_{Xk}U_{i}+v_{Xik},\;k=1,\ldots ,K$$

(2) $$Y_{i}=\theta_{0}+\sum_{k=1}^{K}\theta_{k}X_{ik}+\sum_{j=1}^{p}\alpha_{j}G_{ij}+\gamma_{Y}U_{i}+v_{Yi},$$

where *v_Xik_* and *v_Yi_* are independent error terms with mean zero. Note that the *v_Xik_* are not necessarily independent of each other, and so the risk factors may be correlated via the correlation between these error terms as well as their common association with *U_i_* . We assume that the genetic variants are independent of each other and independent of *U_i_* .We further assume that *p > K*, that each genetic variant is associated with at least one risk factor, and that the *p* × *K* matrix with (*j, k*)th element *β_Xjk_* is of full column rank. This latter assumption ensures that the genetic variant associations with the risk factors are not multicollinear and means that each risk factor is associated with at least one genetic variant. The parameters of interest which we aim to estimate are the *θ_k_*’s, which represent the causal effects of the *k*th risk factor on the outcome.

### 2.2 Instrument validity and pleiotropy

**Figure 1:**
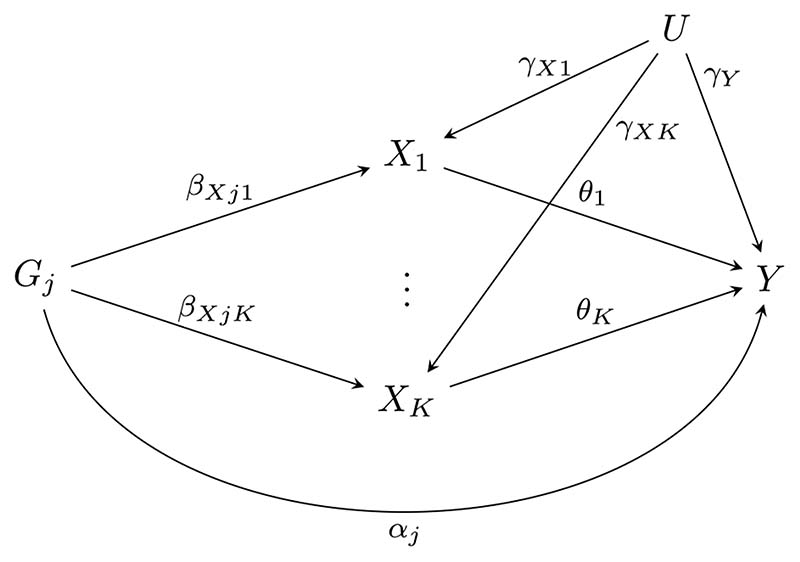
Directed acyclic graph showing the relationship between the *j*th genetic variant (*G_j_*), the risk factors (*X*_1_, …, *X_K_*), confounders (*U*) and the outcome (*Y*)

The relationships between a single genetic variant, the risk factors, confounders and outcome in model (1)-(2) are represented by the directed acyclic graph in Figure 1. For the *j*th genetic variant, pleiotropy is caused by the *α_j_* term. Since the model allows for no direct association between the genetic variant and *U_i_*, *G_j_* is a valid instrument if at least one of *β*_*Xj*1_, …,*β_XjK_* are non-zero and *α_j_* = 0. Note that although the model suggests *α_j_* represents direct effects of the genetic variant on the outcome, it may also represent an association via an unmeasured trait, including via a confounder of the risk factor-outcome relationship.^12^

If *α_j_* = 0 for all *j*, then there is no pleiotropy and all genetic variants are valid instruments. When not all *α_j_’s* are zero, we consider two patterns of pleiotropy. The first is referred to as balanced pleiotropy, which is where the *α_j_’s* are distributed with mean zero. The second is referred to as directional pleiotropy, which is where the *α_j_*’s are distributed with mean not equal to zero. It may also be that most of the *α_j_* are equal to zero but a relatively small number of them are non-zero and possibly large in magnitude. We will refer to these non-zero *α_j_’s* as outliers.

### 2.3 Model identification

If all genetic variants are valid instruments (ie, *α_j_* = 0 for all *j*), the assumptions given in Section 2.1 ensure that the causal effects are identifiable. If some genetic variants are valid and some are invalid, then, following Kang etal,^12^ the parameters *α*_1_, …,*α_p_*,*θ_k_*, …,*Θ_K_* are identifiable if there is a unique solution to

(3) $${\tilde{\beta }}_{Yj}=\alpha_{j}+\theta_{1}{\tilde{\beta }}_{Xj1}+\ldots +\theta_{K}{\tilde{\beta }}_{XjK},$$

*j* = 1, …,*p*, given ${\tilde{\beta }}_{Yj}=E\left(\sum_{i}{\tilde{G}}_{ij}{\tilde{Y}}_{i}\right)/E\left(\sum_{i}{\tilde{G}}_{ij}^{2}\right)$ and ${\tilde{\beta }}_{Xjk}=E\left(\sum_{i}{\tilde{G}}_{ij}{\tilde{X}}_{ik}\right)/E\left(\sum_{i}{\tilde{G}}_{ij}^{2}\right),k=1,\ldots ,K$, *k* = 1, …,*K*, where ${\tilde{G}}_{ij}$, ${\tilde{Y}}_{i}$, and ${\tilde{X}}_{ik}$ are the mean corrected values of *G_ij_*, *Y_i_*, and *X_ik_*, respectively. Let *K* = 1 and consider all subsets of {1, …,*p*} which have the property that ${\tilde{\beta }}_{Yj}=q{\tilde{\beta }}_{Xj1},$ for some constant *q*, for all *j* in the subset. Kang et al^12^ showed that the model parameters are identified if and only if there is no such subset which contains invalid instruments which is as large as, or larger than, the subset which contains all the valid instruments. This is often referred to as the plurality valid assumption. Thus, a sufficient condition for identifiability is that more than half the instruments are valid.

It follows that in the multivariable case, assuming all genetic variant-risk factor association vectors are linearly independent, we require there to be no subset of {1, …,*p*} which satisfies ${\tilde{\beta }}_{Yj}=q_{1}{\tilde{\beta }}_{Xj1}+\ldots +q_{K}{\tilde{\beta }}_{XjK}$for constants *q*_1_, …, *q_K_*, for all *j* in the subset, which is greater than the size of the subset of all valid instruments minus *K*. A sufficient condition for identification is thus that more than (*p* + *K* − 1)/2 genetic variants are valid instruments.

### 2.4 Summary level data

We denote by ${\hat{\beta }}_{Xjk}$ and ${\hat{\beta }}_{Yj}$ the estimates obtained by regressing the *k*th risk factor and outcome, respectively, on the *j*th genetic variant. We have that

$${\hat{\beta }}_{Xjk}=\beta_{Xjk}+\varepsilon_{Xjk}$$

$${\hat{\beta }}_{Yj}=\alpha_{j}+\sum_{k=1}^{K}\beta_{Xjk}\theta_{k}+\varepsilon_{Yj},$$

where var $\left(\varepsilon_{Xjk}\right)=\sigma_{Xjk}^{2}$ and Var $\left(\varepsilon_{Yj}\right)=\sigma_{Yj}^{2}$ In two sample Mendelian randomization the genetic variant-risk factor and genetic variant-outcome associations are estimated in separate samples and so *ε_Xjk_* and *ε_Yj_* are independent for all *j*. If ${\hat{\beta }}_{Xjk}$ and ${\hat{\beta }}_{Xjl}$ and are obtained from separate samples, then *ε_Xjk_* and *ε_xjl_* are independent. Otherwise, the correlation between *ε_Xjk_* and *ε_Xjl_* depends on the correlation between the *k*th and *l*th risk factors. Finally, although in practice they are estimated from data, it is commonly assumed that $\sigma_{Xjk}^{2}$ and $\sigma_{Yj}^{2}$ are known without error for all *j*, *k*.

Although the model assumes that the risk factors and outcome are continuous, categorical traits are possible, and in fact common in practice. In this case, the relevant genetic variant-trait associations are estimated by logistic regression (or ordinal logistic regression, for ordinal variables with more than two categories) and represent the change in log odds ratio of the trait per extra effect allele in the genetic variant.

Note that summary level estimates used for Mendelian randomization are typically unadjusted for other variants (ie, they are computed separately for each variant). In large samples, the differences between the unadjusted estimates and adjusted estimates (ie, those computed using all variants in the same model) will be negligible.

### 2.5 Genetic variant orientation

Each genetic variant can be coded in two ways, depending on which allele is chosen as the effect allele. The choice of effect allele is arbitrary, but will change the sign of the genetic variant-trait associations. Some Mendelian randomization methods may give different results depending on the orientation of the genetic variants. For example, in the single risk factor case, the inverse-variance weighted method^3^ is not affected by genetic variant orientation, but methods which model pleiotropic effects, such as the MR-Egger method^13^ and the lasso-based approach of Rees et al,^14^ are.

In univariable Mendelian randomization, it is conventional to orientate the genetic variants such that an additional copy of the effect allele has a positive association with the risk factor. In the multiple risk factor case, however, this may be done in multiple ways, since forcing positive associations with respect to one risk factor may change the sign of the associations with respect to the others. Rees etal^9^ suggest orientating the genetic variants such that each one has a positive association with the primary risk factor of interest. If there is no single primary risk factor of interest, or as an additional sensitivity analysis, the impact of the orientation may be assessed by repeating the analysis multiple times, re-orientating the genetic variants with respect to each risk factor.

### 2.6 The InSIDE assumption

An assumption that is often required for pleiotropy robust Mendelian randomization is the InSIDE assumption (instrument strength independent of direct effects).^13^ At a ‘population’ level, the InSIDE assumption is that the α*j*’s are independent of each of *β_Xj1_*, …, *β_XjK_*. In a finite sample, we require that the correlation between the sample estimates of the *j* and each of the *β_Xj1_*, …,*β_XjK_*, for the given set of data, is equal to zero. This latter condition will rarely be true in practice, since there will typically be residual correlation due to random variation. If the former is true, however, then this correlation will tend to 0 as the number of instruments increases.

## 3 Methods

We proceed to recall existing methods for multivariable Mendelian randomization in Section 3.1 before introducing new approaches in Section 3.2.

### 3.1 Existing methods for multivariable Mendelian randomization

The multivariable inverse-variance weighted (MVMR-IVW) method^5,15^ fits the multiple linear regression model

(4) $${\hat{\beta }}_{Yj}=\sum_{k=1}^{K}{\hat{\beta }}_{Xjk}\theta_{k}+\varepsilon_{j},$$

*j* = 1, …,*p*, where *ε_j_* is normally distributed with mean zero and variance $\sigma_{Yj}^{2}$ The estimator is obtained using weighted least squares estimation and is thus given by

(5) $$\underset{\theta_{1},\ldots ,\theta_{K}}{\arg\min}\sum_{j=1}^{p}\frac{1}{\sigma_{Yj}^{2}}{\left({\hat{\beta }}_{Yj}-\sum_{k=1}^{K}{\hat{\beta }}_{Xjk}\theta_{k}\right)}^{2}.$$

If all genetic variants are valid instruments, ${\hat{\theta }}_{IVW}$ is a consistent estimator of *θ*. If not all genetic variants are valid instruments, the estimator remains consistent if pleiotropy is balanced and InSIDE is met. Thus, it is sensitive to outliers and directional pleiotropy.

If some of the genetic variants are invalid and pleiotropy is directional, the causal effect can still be consistently estimated using the MVMR-Egger method.^9^ This method fits an intercept term in (4) to account for pleiotropy. That is, we obtain the estimator from

$$\underset{\theta_{0},\theta_{1},\ldots ,\theta_{K}}{\arg\min}\sum_{j=1}^{p}\frac{1}{\sigma_{Yj}^{2}}{\left({\hat{\beta }}_{Yj}-\theta_{0}-\sum_{k=1}^{K}{\hat{\beta }}_{Xjk}\theta_{k}\right)}^{2}.$$

The MVMR-Egger estimator is robust to directional pleiotropy, even when all instruments are invalid. However, it relies on the InSIDE assumption for consistent estimation. Furthermore, it results in lower precision. A final drawback is that it may produce different results depending on the orientation of the genetic variants.

The MR-PRESSO method^16^ has been proposed to handle the case where pleiotropy is balanced but there are outliers. Broadly speaking, the method performs a test based on a heterogeneity measure to identify outliers, which are then removed from the analysis. Although Verbanck et al^16^ has been proposed to handle the case where pleiotropy is balanced but there are outliers. Broadly speaking, the method performs a test based on a heterogeneity measure to identify outliers, which are then removed from the analysis. Although Verbanck et al16 describe the method for the single risk factor case, the authors have also produced a multivariable version, which is a straightforward extension. Specifically, the method computes inverse-variance weighted estimates by leaving out one genetic variant at a time. Letting ${\hat{\theta }}_{1,-j},\ldots ,{\hat{\theta }}_{K,-j}$ be the estimates obtained after leaving out the *j*th genetic variant, it then computes the following quantity, termed the global observed residual sum of squares:

$${\text{RSS}}_{\text{obs}}=\sum_{j=1}^{p}\frac{1}{\sigma_{Yj}^{2}}{\left({\hat{\beta }}_{Yj}-\sum_{k=1}^{K}{\hat{\beta }}_{Xjk}{\hat{\theta }}_{k,-j}\right)}^{2}.$$

This is compared with an expected residual sum of squares, which is computed multiple (*M*) times:

$${\mathrm{RSS}}_{\text{exp}}^{m}=\sum_{j=1}^{p}\frac{1}{\sigma_{Yj}^{2}}{\left({\hat{\beta }}_{Yj}^{\left(m\right)}-\sum_{k=1}^{K}{\hat{\beta }}_{Xjk}^{\left(m\right)}{\hat{\theta }}_{k,-j}\right)}^{2},$$

where ${\hat{\beta }}_{Xjk}^{\left(m\right)},j=1,\ldots ,p,k=1,\ldots ,K$, are drawn from the normal distribution with mean ${\hat{\beta }}_{XjK}$, and variance $\sigma_{Xjk}^{2}$, ${\hat{\beta }}_{Yj}^{\left(m\right)}$, *j* = 1, …,*P*, are drawn the normal distribution with $\sum_{k=1}^{K}{\hat{\beta }}_{Xjk}{\hat{\theta }}_{k,-j}$ and variance $\sigma_{Yj}^{2}$ and *m* = 1, …,*M*. Finally, for each *j*, an empirical *P*-value is computed as

$$\frac{1}{M}\sum_{m=1}^{M}1_{>{\text{RSS}}_{\text{obs}}}\left({\text{RSS}}_{\text{exp}}^{m}\right),$$

where **1** *A*(x) is the indicator function. If the *j*th empirical P-value,multiplied by the number of variants (in order to apply a Bonferroni correction), is greater than the chosen significance level (eg, 0.05), then the respective genetic variant is identified as an outlier. If there are no outliers identified, the estimate obtained is the same as MVMR-IVW. If true outliers are identified and removed, it is expected to reduce the bias and be more efficient than MVMR-IVW. However, the method is not expected to perform well when a large number of genetic variants are pleiotropic, particularly if the pleiotropy is directional.

The methods discussed in this section assume that the genetic variants are strongly associated with each risk factor, conditional on the other risk factors. If this is not the case, estimation may be susceptible to weak instrument bias. Sanderson et al^10^ proposed a conditional F statistic to assess instrument strength, as well as an approach to estimation which provides robustness to weak instruments by adjusting the weights in (5) to incorporate the covariance matrices of the genetic variant-risk factor associations. Furthermore, they proposed including a heterogeneity parameter in the weights to account for balanced pleiotropy. The approach is equivalent to minimizing a Q statistic, and so we refer to it subsequently as MVMR-Q(het).

### 3.2 Proposed pleiotropy robust methods

#### 3.2.1 Robust regression

A natural extension to MVMR-IVW is to use robust regression methods, for example MM-estimation. These methods provide robustness to observations which “contaminate” the data, such as outliers and influential observations (ie, those for which a small change in observed value results in a large change in parameter estimate). A method for performing robust regression in univariable Mendelian randomization is described in Rees et al,^14^ which uses MM-estimation along with Tukey’s bisquare objective function. It is straightforward to extend this approach to the multivariable model: MM-estimation as described by Koller and Stahel^17^ is done in a multivariable setting, and it can be implemented using existing software.

This method of robust regression provides robustness to outliers by effectively capping residuals of a certain magnitude. The approach is thus expected to be robust to pleiotropy when there are a relatively small number of invalid instruments. In this case it should be unbiased and more efficient than MVMR-IVW. However, it may not perform well if there are a relatively large number of invalid instruments.

#### 3.2.2 Median based estimation

An alternative approach to robust regression is to use least absolute deviations regression. That is, we estimate *θ* by

(6) $$\underset{\theta_{1},\ldots ,\theta_{K}}{\arg\min}\sum_{j=1}^{p}\frac{1}{\sigma_{Yj}^{2}}\left|{\hat{\beta }}_{Yj}-\sum_{k=1}^{K}{\hat{\beta }}_{Xjk}\theta_{k}\right|.$$

Least absolute deviations regression is a special case of quantile regression which estimates the 50th percent ile. Thus, (6) is easily computed using techniques developed for quantile regression.^18^ Since ${\hat{\beta }}_{Yj}$ and ${\hat{\beta }}_{Xjk}$ are continuous, (6) has a unique solution with probability one.

Similar to robust regression, least absolute deviations regression is less affected by outliers than least squares regression. It is not robust to influential observations, as robust regression is. However, it may be expected to perform better when the distribution of the ${\hat{\beta }}_{Yj}$ are not symmetric. That is, it also provides robustness to directional pleiotropy. When *K* = 1, the estimator obtained using least absolute deviations regression is equivalent to the weighted median estimator for univariable Mendelian randomization proposed by Bowden et al^19^ with weights given by $\left|{\hat{\beta }}_{Xj1}\right|/\sigma_{Yj}^{2}$ (note that, strictly speaking, it is equivalent to the weighted empirical distribution method described in the supplementary material to that paper). The least absolute deviations regression approach can thus be thought of as a natural extension of median-based methods to the multivariable setting. We therefore refer to the method as MVMR-Median.

A disadvantage of least absolute deviations regression is that we lose the asymptotic theory of least squares estimation which leads to easy to compute and accurate standard errors for use, for example, in inference. Confidence intervals are typically produced using a rank inversion technique, or via resampling methods (see, eg, Tarr^20^). Here we take advantage of the fact that we know the distribution of the genetic variant-trait associations, and implement a parametric bootstrap procedure, as follows. For each genetic variant, a genetic variant-outcome association is drawn from the normal distribution with mean ${\hat{\beta }}_{Yj}$ and variance $\sigma_{Yj}^{2}$ and genetic variant-risk factor associations are drawn from the multivariate normal distribution with mean ${\left({\hat{\beta }}_{Xj1},\ldots ,{\hat{\beta }}_{XjK}\right)}^{\prime }$ and covariance matrix diag $\left(\sigma_{Xj1}^{2},\ldots ,\sigma_{XjK}^{2}\right)$. The estimated standard error is the standard deviation of the estimates computed from multiple replications of this sampling. This approach does not take into account correlation between the risk factors, however the simulation results presented in Section 4 and the supplementary material show it still performs well in the correlated risk factor case. Finally, we note that an application of multivariable Mendelian randomization using quantile regression, and a rank inversion technique for producing confidence intervals, has been previously performed by Gill et al^21^ in a study which examined the effects of education on coronary heart disease.

#### 3.2.3 Regularization methods

Under the assumption that some of the *α_j_’s* are zero and some are not, regularization methods for univariable Mendelian randomization have been proposed which include an intercept term for each genetic variant in the least squares equations (5) and then apply lasso-type penalization to these terms. The penalization tends to shrink the intercept terms corresponding to the valid instruments toward zero. It thus accounts for the pleiotropy caused by invalid instruments, without the loss of power and need for the InSIDE assumption of Egger regression. The approach was first proposed by Kang etal^12^ in the individual level setting, and followed up by Windmeijer etal.^22^ Rees etal^14^ developed a regularization approach using summary level data.

In the multivariable setting we propose using

(7) $$\underset{\theta_{01},\ldots ,\theta_{0p},\theta_{1},\ldots ,\theta_{K}}{\arg\min}\overset{p}{\underset{j=1}{\sum^{+}}}\frac{1}{\sigma_{Yj}^{2}}{\left({\hat{\beta }}_{Yj}-\theta_{0j}-\overset{k=1}{\underset{K}{\sum^{+}}}{\hat{\beta }}_{Xjk}\theta_{k}\right)}^{2}+\lambda \overset{p}{\underset{j=1}{\sum^{+}}}++\left|\theta_{0j}\right|,$$

for some tuning parameter *λ* > 0. This is not a standard lasso problem, since not all regression parameters are being penalized. However, the parameter estimates can be easily computed using the algorithm given in Section S1 of the supplementary material, which uses only standard regression and lasso procedures. The tuning parameter controls the level of sparsity. The larger the value, the fewer genetic variants will be identified as invalid, and the estimate will approach the MVMR-IVW estimate. A data driven approach to choosing the tuning parameter is to use the heterogeneity stopping rule described by Rees et al.^14^

The lasso penalty will shrink some *θ_0_j’s* exactly to zero, thus identifying the corresponding genetic variants as being valid instruments. A post-lasso estimator takes the genetic variants identified as valid and fits a standard MVMR-IVW model using only these variants. Post-lasso estimators have been advocated by, for example, Efron et al^23^ and Belloni etal,^24^ in order to avoid bias caused by the shrinkage of parameter estimates. The lasso algorithm is thus effectively used as a model selection technique.

A limitation of regularization techniques generally is the inability to compute accurate standard errors. We can compute standard errors for the post-lasso estimator using a random effects model^15^ in the post-selection regression. However, this ignores the uncertainty associated with the model selection event. As a result, the standard errors are likely to be too small, and the type I error rate inflated. We examine the effect of this in the simulation study presented in Section 4. A way around this is to use a three sample approach: here, a set of genetic variant-trait associations is used for performing the MVMR-Lasso procedure which are taken from a sample (or samples) which are independent of those from which the genetic variant-trait associations used for the post-lasso estimator are taken. In this way, the model selection and estimation procedures are independent and the correct type I error rate will be retained.^25,26^ Although this restricts the potential scope for analyses, since multiple independent samples of genetic variant associations with the traits of interest are required, there are still a number of risk factor-outcome combinations which can be studied given the wide variety of GWAS results which are publicly available. Another promising development in the univariable setting is the use of a selective inference approach, which aims to derive a conditional distribution of the estimator given the model selection event.^27^

One final point to note is that the solution to (7) may be different depending on the orientation of the genetic variants. Following the convention used when performing MVMR-Egger, we propose orientating the genetic variants such that the genetic variant associations with the primary risk factor of interest are all positive.

## 4 Simulations

We conducted a simulation study to compare the performance of the methods described in the previous section under scenarios with different amounts and types of pleiotropy. We simulated from model (1)-(2) with the intercepts set to zero, *p* = 100 genetic variants, *K* = 4 risk factors, *n* = 20000, *γ_Xj_* = 1/*K*, *γ_Y_* = 1, *β_Xjk_ ~* Uniform (0,0.1), *G_ij_* ~ Binomial (2, 0.3),

$$U_{i}=\sum_{j=1}^{p}\delta_{j}G_{ij}+w_{i}$$

and *v_Xi_*1, …, *v_XiK_*, *v_Yi_*, *w_i_* ~ N (0, 1), independently. These parameter values give *R* ^2^ statistics (ie, the proportion of the variance in each risk factor explained by the genetic variants) of approximately 12%. Two sets of values for the causal effects were considered: in the first, *θ*_1_ = 0.2, *θ*_2_ = 0.1, *θ*_3_ = 0.3, *θ*_4_ = 0.4; in the second, *θ*_1_ = 0,*θ*_2_ = -0.1, *θ*_3_ = 0.1, *θ*_4_ = 0.2. Four scenarios were considered with different patterns of pleiotropy. For each scenario either 10%, 30%, 50% or 70% of genetic variants were invalid.

1. Balanced pleiotropy and InSIDE assumption met: All *δ_j_*’s were set to zero and the *α_j_*’s corresponding to invalid instruments were generated from the *N* (0, 0.2^2^) distribution.
2. Directional pleiotropy and InSIDE assumption met: All *δ_j_*’s were set to zero and the *α_j_*’s corresponding to invalid instruments were generated from the *N* (0.1,0.2^2^) distribution.
3. Directional pleiotropy and InSIDE assumption violated: The *α_j_*’s corresponding to the invalid instruments were generated from the *N* (0,0.2^2^) distribution and the *δ_j_*’ s corresponding to the invalid instruments were generated from the Uniform (0, 0.1) distribution.
4. Balanced pleiotropy and InSIDE assumption violated: The *α_j_*’s corresponding to the invalid instruments were generated from the *N* (0,0.2^2^) distribution and the *δ_j_*’s corresponding to the invalid instruments were generated from the Uniform (-0.05, 0.05) distribution.

In scenarios 3 and 4, the InSIDE assumption is violated because of the direct association of the genetic variants with the confounder. This creates a pleiotropic effect of the genetic variants on the outcome which is not independent of the effect of the genetic variants on the risk factors due to the association of the confounder with the risk factors.^11^ It should also be noted that the overall pleiotropic effect in Scenario 3 is directional because the *δ_j_* parameters have non-zero mean.

**Figure 2:**
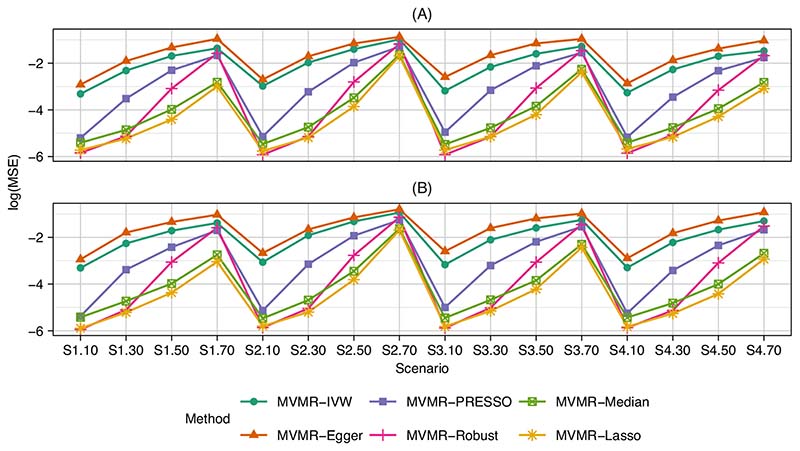
Logarithm of the mean squared errors for each scenario (S1, S2, S3, and S4) and proportion of invalid genetic variants (10%, 30%, 50% or 70%), where (a) *θ*_1_ = 0.2 and (b) *θ_1_* = 0 [Colour figure can be viewed at wileyonlinelibrary.com]

For each scenario, level of pleiotropy, and set of *θ*_*k*_ values, the simulations were replicated 1000 times. For each replication, the genetic variant-trait association estimates and their standard errors were computed from the individual level data using simple linear regression with an intercept. The causal effects were then estimated using the methods described in Section 3. The parameter of interest which we report on was the causal effect of the first risk factor on the outcome (ie, for the first set of *θ*_*k*_ values, there is a true causal effect, and for the second set of values there is no causal effect). The mean, standard deviation of estimates, mean standard error and power/type I error rate, at the 0.05 significance level, are shown in Tables 1 and 2. The log of the mean squared errors across all scenarios are shown in Figure 2. Note that the mean conditional F statistic for the first risk factor was between 6.3 and 6.6 across the various scenarios. Also note that here the MVMR-Lasso method refers to the two sample post-lasso estimator (ie, with the estimate computed from the same samples that the instruments were selected in).

All methods performed well in terms of bias when there was balanced pleiotropy. The MVMR-IVW and MVMR-Egger methods were biased when pleiotropy was directional, increasing as the proportion of pleiotropy increased. These methods were also less precise than all other methods, with the largest standard deviations of estimates, and were very low powered. In theory, MVMR-Egger should be robust to directional pleiotropy when InSIDE is met. However, there was a fair amount of bias from this method in Scenario 2 when these conditions are satisfied. Given that the conditional F statistics were fairly low, a possible explanation is that the bias is due to weak instruments, which this method is particularly susceptible to. Some evidence supporting this explanation was provided in a supplementary simulation study, where the instrument strength was increased and the bias from MVMR-Egger was reduced to close to zero. This supplementary simulation study is discussed in more detail later in this section.

MVMR-Robust outperformed MVMR-PRESSO in all scenarios up to 50% pleiotropy with lower bias, more precision and correct type I error rates. Notably, MVMR-Robust had type I error rates at or below the significance level across all scenarios, even with 70% pleiotropy. MVMR-PRESSO had low bias at the lower level of pleiotropy, but did not perform well with moderate or high amounts. MVMR-Lasso was generally the most precise estimate: it had similar mean squared error to MVMR-Robust at 10% pleiotropy, but retained its performance in this regard at the higher levels of pleiotropy also. Similarly, it had comparable power to MVMR-Robust at 10% pleiotropy, but did much better as the proportion of pleiotropy increased. As expected, MVMR-Lasso had inflated type I error rates. MVMR-Median had comparable bias to MVMR-Lasso across all levels of pleiotropy. It was less precise and lower powered than MVMR-Lasso, but had type I error rates closer to the significance level. In terms of mean squared error, MVMR-Median was bettered uniformly across all scenarios only by MVMR-Lasso. As a further analysis, in Figure S1 in the supplementary material, we compare the mean squared errors with those from the estimates of the causal effect of the fourth risk factor, that is, where the true causal effect is θ4 = 0.4. The average proportion of variation in the outcome explained by the fourth risk factor is approximately 19%, compared with approximately 11% for the first risk factor. However, with the exception of MVMR-Egger, the results are almost identical across all scenarios. Finally, we considered the performance of the methods in estimating the full *θ*. vector by comparing the mean squared error defined as the mean, across all replications, of $\sum_{k=1}^{4}{\left({\hat{\theta }}_{j}-\theta_{j}\right)}^{2}$, where ${\hat{\theta }}_{j}$ is the estimate of *θj*. As shown in Figure S2, the relative performance of the methods is in line with those in Figure 2.

The simulations were repeated for the cases where there were fewer instruments (*p* = 20, with the distribution of the *β_Xjk_* parameters adjusted to retain similar *R^2^* values), where the risk factors were correlated by setting cor (*v_Xik_, v_Xil_*) = 0.5 for all *k* ≠ *l*, and where the genetic variant-trait associations were all estimated from the same sample (one sample Mendelian randomization). For brevity, supplementary analyses were performed over Scenarios 1-3 and for the 10%-50% pleiotropy cases. The results are shown in Tables S1-S6 and Figures S3-S5. In each case, the results followed a similar pattern as before in terms of comparative performance of the different methods. In the fewer instruments case, there was slightly higher mean squared error across the board, which would be expected with fewer instruments, but the differences were not great. It is also notable that the bias from the MVMR-Egger method in Scenario 2 was close to zero, in contrast to the primary simulation results shown in Tables 1 and 2. As noted earlier, this supports the assertion that the bias from MVMR-Egger in this scenario in the primary simulations was at least partly due to weak instruments, since the conditional F statistics were much higher (between 22.9 and 23.8) in the fewer instruments case. A further note is that the results from MVMR-Median in the correlated risk factor case are in line with those in the uncorrelated risk factor case. This suggests that the parametric bootstrap procedure (which effectively ignores risk factor correlation) is robust to the case where the risk factors are correlated.

Three further supplementary analyses were performed to examine various aspects of the proposed methods. The first compared the parametric bootstrap procedure for computing confidence intervals for MVMR-Median to two alternative approaches: a nonparametric bootstrap procedure, where the genetic variants were sampled, with replacement, 1000 times to estimate an empirical distribution function of the estimate; and a rank inversion technique as described by Koenker.^28^ Table S7 shows the coverage and confidence interval width for each method across each scenario. The parametric bootstrap gave narrower confidence intervals, on average, than the other methods in all cases. In almost all cases, the parametric bootstrap procedure also had better coverage,with the only exceptions being that the nonparametric bootstrap had slightly higher coverage in some of the scenarios with 50% pleiotropy.

Another supplementary analysis considered the case where there was mediation of the effect of the first risk factor on the outcome by another risk factor (see Figure S6). In these scenarios, the multivariable methods estimate the direct effect of the first risk factor on the outcome, rather than the total effect which includes the mediated effect via the second risk factor. The results of these simulations are shown in Tables S8 and S9, and demonstrate that the proposed methods extend to the case where there are causal pathways among the risk factors.

The final supplementary analysis evaluated how well MVMR-Lasso correctly selected the instruments as valid or invalid. This is discussed in detail in Section S2.1. Although the ability to select invalid instruments is an interesting aspect of this method, we stress that instrument selection is not the primary aim of this approach, since the lasso procedure is trained to minimize heterogeneity. However, the results of this analysis show that the method is generally reliable in identifying whether instruments are valid or not. In particular, in the primary simulation scenarios, any genetic variant identified as invalid was almost always truly invalid, and truly valid instruments were almost always identified as valid.

## 5 Applied Example: The Causal Effect Of Intelligence, Education And Household Income On Alzheimer’S Disease

In this section we consider an applied example looking at the causal effects of intelligence, years of education and household income on Alzheimer’s disease. The effects of intelligence and years of education on health outcomes have been studied by Davies etal^29^ and Anderson etal.^30^ A multivariable approach is important in this case since intelligence and years of education are highly correlated. Anderson et al^30^ used both univariable and multivariable Mendelian randomization with intelligence and years of education as risk factors and Alzheimer’s disease as outcome. Their results from applying the univariable inverse-variance weighted method (MR-IVW) with each risk factor separately suggest that both intelligence and years of education have a protective effect on Alzheimer’s disease. However, when both risk factors are included in a multivariable model, using MVMR-IVW, the effect of years of education, independent of intelligence, shifts toward the null. The implication is that years of education only has a causal effect on the odds ratio of Alzheimer’s disease via its effect on intelligence. Here, we reconsider this example and include household income as an extra risk factor.

Genetic variant associations with intelligence and years of education are taken from the GWAS of Hill et al^31^ and Okbay et al,^32^ respectively. By clumping the combined list of genetic variants which associate with each risk factor at the genome wide significance level, Davies et al^29^ arrived at a list of 219 independent genetic variants to be used as instruments in multivariable Mendelian randomization analyses. We obtained the associations between these genetic variants and household income from the UK Biobank (sourced from http://www.nealelab.is/uk-biobank/). Note that household income is an ordinal categorical variable, and so the genetic variant associations represent the increase in log odds of being in a higher income category per extra effect allele. Genetic variant associations with Alzheimer’s disease were obtained from the GWAS of Lambert etal.^33^ In total, 213 of the genetic variants used by Davies etal^29^ were available in both of the household income and Alzheimer’s disease datasets, and we used these as instruments in our analysis.

Note that the genetic variant associations with both intelligence and years of education were all in the same direction, and so they were orientated in our analysis to be all positive with respect to these traits.

**Figure 3:**
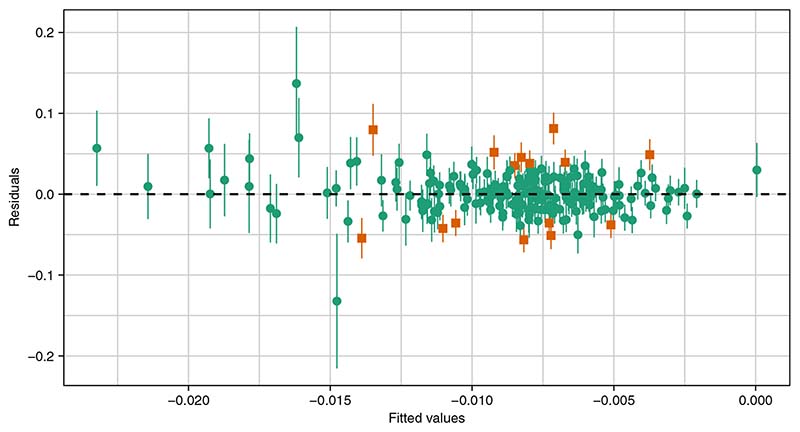
Residuals vs fitted values obtained from applying the MVMR-IVW method to the applied example studying the causal effects of intelligence, years of education and household income on Alzheimer’s disease. The vertical error bars indicate *±σ_Y_j* for each genetic variant. The orange box shaped points indicate the genetic variants identified as pleiotropic by the MVMR-Lasso method [Colour figure can be viewed at wileyonlinelibrary.com]

**Figure 4:**
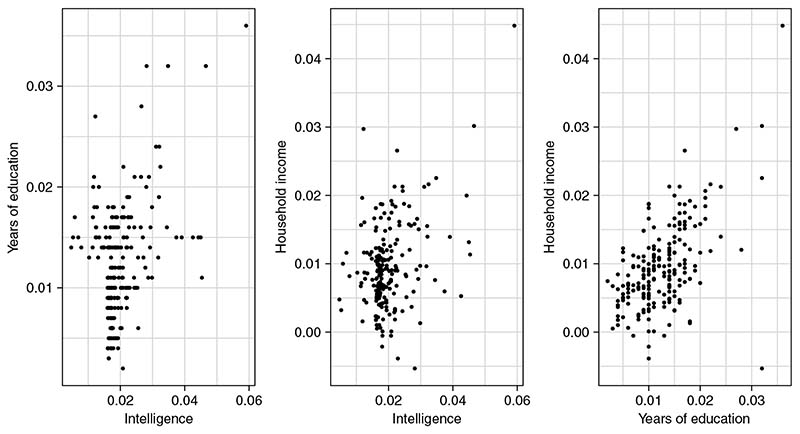
Scatterplots of each pair of genetic variant associations with intelligence, years of education and household income

Figure 3 shows a plot of the residuals vs fitted values after fitting the MVMR-IVW model to the data. The vertical error bars indicate *±σ_yj_* for each genetic variant. The plot provides a way of visualizing heterogeneity in the multivariable setting, similar to the scatterplots of ${\hat{\beta }}_{Xj}$ against ${\hat{\beta }}_{Yj}$ commonly used in the univariable case. Although there is little evidence of directional pleiotropy, there may be some outliers. Figure 4 shows scatterplots of each pair of genetic variant-risk factor associations. There appears to be reasonably strong correlation between the genetic variant associations with years of education and household income, and low to moderate correlation between the other two pairs of associations. A large degree of multicollinearity can cause numerical instability in the estimates, and perfect multicollinearity suggests that the full rank condition given in Section 2.1 may be violated. We can assess the degree of multicollinearity using the condition number of the matrix of genetic association estimates with the risk factors, where a higher value of the condition number indicates increased multicollinearity (see Section S3 for further details). The condition number of 6.8 is below the typically recommended threshold of 30 where numerical instability due to multicollinearity is considered a concern.^34^

We assessed instrument strength in the univariable setting using the mean F statistic across the genetic variants, and in the multivariable setting using the conditional F statistic (see Table 3). When taking each risk factor separately, the mean F statistics are all over 10, suggesting that the genetic variants are strong instruments. This is expected since the instruments were selected according to genome-wide significance and since the risk factors are moderately to strongly correlated. However, the conditional F statistics are much lower which suggests that the instruments are individually weak conditional on the others. In light of this, along with the multivariable approaches used in the simulation study reported in Section 4, we also applied the MVMR-Q(het) method in order to provide a sensitivity analysis which is robust to weak instruments.

**Figure 5:**
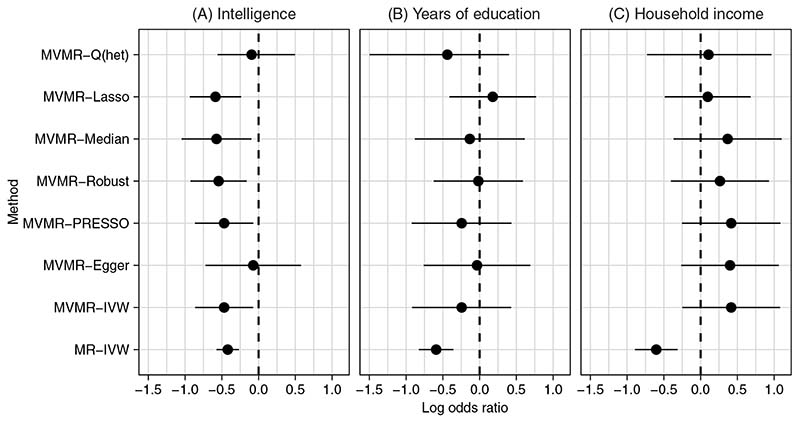
Log causal odds ratio for Alzheimer’s disease point estimate and 95% confidence interval per one standard deviation increase in: A, Intelligence; B, Years of education and; C, Per unit increase in log odds ratio for a higher household income bracket

Figure 5A-C and Table 3 show the results of applying MR-IVW to estimate the causal effect of each risk factor separately on the odds ratio of Alzheimer’s disease, as well as each of the multivariable methods with all three risk factors included. The univariable analyses suggest that intelligence and years of education both have a protective effect on Alzheimer’s disease, in line with the results of Anderson et al.^30^ The estimated log causal odds ratio of Alzheimer’s disease per one standard deviation increase in intelligence is -4.20 (95% CI -0.57 to -0.27), and per one standard deviation increase in years of education is -0.59 (95% CI -0.83 to -0.36). The estimated log causal odds ratio of Alzheimer’s disease per unit increase in log odds ratio for a higher household income bracket is -0.60 (95% CI -0.89 to -0.31). Using the MVMR-IVW model, the estimates of the log causal odds ratio from both years of education and household income attenuated to the null, with 95% confidence intervals overlapping zero. The multivariable model however still suggests a protective effect from intelligence, with an estimated odds ratio of -0.47 (95% CI -0.86 to -0.07).

The pleiotropy robust multivariable methods gave results which were broadly consistent with the MVMR-IVW results (see Table 3). The MVMR-Egger and MVMR-Q(het) methods suggested a null causal effect of intelligence on Alzheimer’s disease, however their point estimates are still in the same direction and all other methods were in line with MVMR-IVW. Note that the MR-PRESSO outlier test did not detect any outliers, but the MVMR-Lasso method identified 15 genetic variants as pleiotropic,which were removed before computing the post-lasso estimator. These genetic variants are indicated in Figure 3. Interestingly, the post-lasso estimate for the causal effect of years of education on Alzheimer’s disease was positive, where as all other estimates of this effect were negative. However, the confidence interval still included the null. Note also that the estimate from MVMR-Q(het) for the causal effect of years of education was some what different to the other pleiotropy robust methods, however the confidence interval was very wide and overlapped with all other point estimates.

The consistency of the findings give strength to the assertion that intelligence has a causally protective effect on Alzheimer’s disease, conditional on years of education and household income. However, there is no evidence of a direct effect of years of education or household income on Alzheimer’s disease. There are two potential explanations for why years of education and household income would appear to have an effect in the univariable analysis but not in the multivariable analysis. One is that these risk factors affect Alzheimer’s disease but only via their association with intelligence. That is, that intelligence is a mediator of the effect of these risk factors on Alzheimer’s disease. The other explanation is that the genetic variants that affect education and household income have pleiotropic effects, potentially via intelligence. It is also possible that both are true (ie, that there is both pleiotropic effects and mediation via intelligence).

## 6 Discussion

In this article we have presented methods for performing multivariable Mendelian randomization which are robust to pleiotropy. Existing methods either allow for invalidity at the cost of low precision and the InSIDE assumption (MVMR-Egger), orwere developed for the casewhere pleiotropy is balanced and there are a relatively small number of outliers (MVMR-PRESSO). We have considered methods which can handle higher proportions of invalidity and directional pleiotropy.

When there is evidence of relatively few invalid instruments, MVMR-Robust was shown to outperform MVMR-PRESSO in all scenarios with pleiotropy up to 50%. MVMR-Lasso, another method which aims to identify and down weight outliers, performed best overall in terms of mean squared error, even when half of the genetic variants were in valid instruments and pleiotropy was directional. Although type I error rates were inflated,this can be mitigated when a three sample approach is possible. MVMR-Median was shown to perform almost as well as MVMR-Lasso in terms of mean squared error, and retained correct type I error rates at higher levels of pleiotropy. Although all methods showed considerable bias in the settings with high levels of directional pleiotropy (ie, where 50% or 70% of instruments were invalid), our newly proposed methods still out performed existing methods across the various metrics considered in these extreme scenarios. Furthermore, MVMR-Robust retains correct type I error rates even with very high levels of pleiotropy. As demonstrated in the applied example,a plot of the residuals versus fitted values from the MVMR-IVW method can be used to visualize potential outliers and pleiotropy, and to help determine the most appropriate choice of robust method.

Although our initial model assumes no causal pathways between the risk factors, the methods are applicable if there are such pathways, as demonstrated by the supplementary simulation analyses. The only difference in this case is in the interpretation of the causal parameters. In the presence of mediation, the estimates represent the direct effects of the risk factors on the outcome, excluding any indirect effects via the mediators. There is existing literature which examines mediation analysis in the Mendelian randomization setting.^35-37^

A potential extension to MVMR-Lasso is to consider different penalty terms for each genetic variant. Although different penalty weighting schemes did not significantly affect our simulation results, this is an interesting area to consider for future research, for example if the association estimates have considerably different levels of precision. A potential extension could be along the lines of the adaptive lasso approach of Windmeijer et al.^22^

The work has some limitations in the modeling assumptions made, in particular of the linearity and homogeneity (ie, no effect modification) of the effects of the risk factors on the outcome. Furthermore, although we can handle non-continuous traits via the use of logistic regression to produce summary statistics, this may cause bias in the causal effect estimates due to the non-collapsibility of the odds ratio. Nonetheless, violations of these assumptions tend to attenuate causal effect estimates toward the null.^11,38^

Another limitation is the assumption of no measurement error of the genetic variant-risk factor associations, equivalent to assuming *σ_X_j_k_* = 0 for all *j*, *k*. This is a common assumption in Mendelian randomization analyses, and is justified by the very large sample sizes that these associations are typically estimated in, in contrast to the genetic variant-outcome associations which may be estimated using a relatively small number of cases vs controls. Provided the genetic variants strongly predict each risk factor, conditional on all the other risk factors, this assumption will have little influence on the analysis. Otherwise, the resultsmay be subject to weak instrument bias. In practice, weak instrument bias is mitigated by selecting a set of genetic variants which associatewith the risk factors according to some threshold related to, for example, F statistics or P-values. Furthermore, the approach of Sanderson et al^10^ provides a sensitivity analysis in case there is still a suspicion of weak instruments, as demonstrated in our applied example. Although in univariable Mendelian randomization measurement error will bias causal effect estimates toward the null,^39^ this will not necessarily be the case in the multivariable setting. Assessing the impact of measurement error in the multiple risk factor case, and how to account for this, is an active area of research.

In summary, the methods we have presented provide new ways for performing Mendelian randomization with multiple risk factors which are robust to different forms of pleiotropy. Each has advantages when applied to specific scenarios. Together with MVMR-Egger, these methods provide a suite of sensitivity analyses for multivariable Mendelian randomization.

## 7 Software

R code for performing the methods described in this article, and for reproducing the simulation results, can be found at https://github.com/aj-grant/robust-mvmr. Existing Mendelian randomization methods were implemented using the following R packages: Mendelian Randomization^40^ (MR-IVW, MVMR-IVW, MVMR-Egger), MRPRESSO^16^ (MVMR-PRESSO) and MVMR^6^ (MVMR-Q(het)). Existing packages which were used in the implementation of the newly proposed methods include: robustbase^41^ (MVMR-Robust), quantreg^42^ (MVMR-Median) and glmnet^43^ (MVMR-Lasso).

## Supplementary Material

Supplementary File

## Figures and Tables

**Table 1:** Mean and standard deviation (SD) of estimates, mean standard error (SE) and power when *θ_γ_* = 0.2

Method	10% invalid				30% invalid				50% invalid				70% invalid			
	Mean	SD	SE	Power	Mean	SD	SE	Power	Mea	SD	SE	Power	Mean	SD	SE	Power
	Scenario 1: Balanced pleiotropy, InSIDE met															
MVMR-IVW	0.201	0.191	0.185	0.222	0.202	0.314	0.321	0.094	0.198	0.430	0.415	0.089	0.228	0.506	0.490	0.088
MVMR-Egger	0.207	0.233	0.226	0.190	0.219	0.385	0.391	0.084	0.207	0.515	0.502	0.074	0.232	0.619	0.596	0.073
MVMR-PRESSO	0.206	0.074	0.067	0.824	0.201	0.172	0.141	0.373	0.186	0.316	0.221	0.243	0.246	0.433	0.304	0.223
MVMR-Robust	0.205	0.054	0.055	0.958	0.203	0.077	0.078	0.735	0.186	0.214	0.235	0.128	0.234	0.452	0.456	0.087
MVMR-Median	0.206	0.067	0.082	0.744	0.202	0.088	0.100	0.528	0.197	0.137	0.127	0.367	0.214	0.244	0.177	0.299
MVMR-Lasso	0.205	0.057	0.058	0.934	0.204	0.073	0.068	0.830	0.199	0.110	0.083	0.652	0.213	0.224	0.107	0.543
	Scenario 2: Directional pleiotropy, InSIDE met															
MVMR-IVW	0.256	0.219	0.206	0.272	0.332	0.349	0.350	0.171	0.416	0.448	0.442	0.159	0.538	0.513	0.507	0.189
MVMR-Egger	0.240	0.257	0.251	0.192	0.267	0.421	0.424	0.105	0.300	0.553	0.536	0.086	0.374	0.623	0.615	0.101
MVMR-PRESSO	0.208	0.076	0.070	0.837	0.239	0.196	0.157	0.390	0.334	0.347	0.253	0.313	0.455	0.458	0.338	0.340
MVMR-Robust	0.203	0.052	0.055	0.963	0.201	0.077	0.080	0.733	0.256	0.240	0.264	0.144	0.471	0.484	0.490	0.143
MVMR-Median	0.207	0.065	0.084	0.733	0.214	0.093	0.105	0.543	0.247	0.169	0.142	0.425	0.337	0.419	0.225	0.400
MVMR-Lasso	0.204	0.056	0.059	0.939	0.205	0.075	0.071	0.809	0.225	0.143	0.089	0.661	0.310	0.417	0.130	0.625
	Scenario 3: Directional pleiotropy, InSIDE violated															
MVMR-IVW	0.238	0.200	0.192	0.276	0.283	0.329	0.329	0.132	0.313	0.436	0.419	0.121	0.357	0.503	0.492	0.122
MVMR-Egger	0.276	0.263	0.231	0.266	0.352	0.410	0.390	0.155	0.385	0.531	0.491	0.145	0.409	0.582	0.569	0.123
MVMR-PRESSO	0.210	0.083	0.074	0.800	0.245	0.201	0.164	0.387	0.285	0.338	0.258	0.270	0.322	0.445	0.338	0.250
MVMR-Robust	0.203	0.052	0.056	0.963	0.205	0.076	0.079	0.748	0.230	0.214	0.239	0.153	0.326	0.464	0.464	0.119
MVMR-Median	0.206	0.064	0.083	0.763	0.214	0.091	0.102	0.570	0.225	0.144	0.133	0.417	0.264	0.317	0.195	0.343
MVMR-Lasso	0.204	0.057	0.059	0.947	0.206	0.076	0.069	0.818	0.213	0.122	0.085	0.657	0.256	0.298	0.116	0.587
	Scenario 4: Balanced pleiotropy, InSIDE violated															
MVMR-IVW	0.208	0.196	0.188	0.233	0.206	0.321	0.323	0.099	0.212	0.427	0.417	0.088	0.224	0.477	0.494	0.067
MVMR-Egger	0.225	0.237	0.228	0.194	0.238	0.390	0.392	0.095	0.245	0.501	0.506	0.077	0.258	0.594	0.600	0.074
MVMR-PRESSO	0.204	0.075	0.068	0.828	0.206	0.178	0.140	0.413	0.207	0.313	0.226	0.248	0.230	0.415	0.303	0.203
MVMR-Robust	0.204	0.053	0.055	0.972	0.203	0.079	0.078	0.739	0.195	0.207	0.234	0.141	0.226	0.432	0.464	0.071
MVMR-Median	0.204	0.067	0.082	0.741	0.201	0.093	0.100	0.548	0.203	0.139	0.127	0.376	0.215	0.244	0.178	0.303
MVMR-Lasso	0.204	0.059	0.058	0.933	0.204	0.076	0.068	0.830	0.204	0.117	0.083	0.645	0.208	0.214	0.108	0.520

**Table 2:** Mean and standard deviation (SD) of estimates, mean standard error (SE) and type I error rate when *θ_γ_ =*; 0

Method	10% invalid				30% invalid				50% invalid				70% invalid			
	Mean	SD	SE	Type I	Mean	SD	SE	Type I	Mean	SD	SE	Type I	Mea	SD	SE	Type I
	Scenario 1: Balanced pleiotropy, InSIDE met															
MVMR-ΓVW	0.003	0.191	0.187	0.054	0.014	0.323	0.321	0.045	0.016	0.424	0.412	0.053	0.020	0.500	0.487	0.052
MVMR-Egger	0.015	0.229	0.228	0.047	0.022	0.408	0.391	0.054	0.017	0.511	0.501	0.055	0.038	0.597	0.593	0.050
MVMR-PRESSO	0.003	0.068	0.064	0.053	0.003	0.184	0.136	0.098	−0.003	0.298	0.214	0.100	0.023	0.427	0.296	0.137
MVMR-Robust	0.002	0.051	0.051	0.053	0.000	0.078	0.078	0.049	−0.001	0.217	0.235	0.028	0.021	0.453	0.455	0.049
MVMR-Median	0.003	0.066	0.071	0.038	0.006	0.094	0.088	0.054	0.001	0.137	0.114	0.093	0.014	0.253	0.165	0.168
MVMR-Lasso	0.002	0.053	0.051	0.064	0.002	0.074	0.061	0.096	0.002	0.112	0.075	0.171	0.015	0.218	0.101	0.349
	Scenario 2: Directional pleiotropy, InSIDE met															
MVMR-ΓVW	0.044	0.212	0.206	0.065	0.123	0.364	0.350	0.070	0.253	0.452	0.441	0.093	0.320	0.538	0.508	0.115
MVMR-Egger	0.026	0.262	0.250	0.058	0.051	0.434	0.425	0.059	0.123	0.551	0.535	0.068	0.174	0.649	0.616	0.072
MVMR-PRESSO	0.007	0.077	0.067	0.064	0.042	0.203	0.157	0.088	0.152	0.350	0.245	0.173	0.238	0.477	0.334	0.200
MVMR-Robust	0.003	0.053	0.052	0.072	0.005	0.080	0.079	0.050	0.070	0.240	0.266	0.023	0.245	0.511	0.496	0.080
MVMR-Median	0.006	0.065	0.072	0.026	0.019	0.094	0.092	0.060	0.056	0.169	0.129	0.126	0.149	0.417	0.214	0.293
MVMR-Lasso	0.003	0.055	0.052	0.063	0.006	0.074	0.063	0.093	0.033	0.145	0.082	0.233	0.131	0.410	0.122	0.502
	Scenario 3: Directional pleiotropy, InSIDE violated															
MVMR-ΓVW	0.043	0.201	0.191	0.057	0.094	0.336	0.330	0.079	0.131	0.431	0.419	0.077	0.161	0.510	0.493	0.069
MVMR-Egger	0.080	0.261	0.230	0.082	0.164	0.418	0.391	0.099	0.192	0.518	0.491	0.088	0.178	0.586	0.571	0.064
MVMR-PRESSO	0.013	0.081	0.070	0.068	0.037	0.198	0.158	0.081	0.081	0.325	0.249	0.115	0.129	0.445	0.332	0.125
MVMR-Robust	0.008	0.052	0.051	0.057	0.013	0.080	0.079	0.051	0.028	0.215	0.241	0.024	0.126	0.468	0.466	0.057
MVMR-Median	0.011	0.065	0.072	0.030	0.019	0.095	0.090	0.066	0.025	0.144	0.120	0.104	0.075	0.308	0.180	0.210
MVMR-Lasso	0.008	0.054	0.051	0.068	0.013	0.075	0.062	0.099	0.014	0.120	0.077	0.183	0.059	0.290	0.107	0.396
	Scenario 4: Balanced pleiotropy, InSIDE violated															
MVMR-ΓVW	0.007	0.192	0.188	0.048	0.017	0.330	0.322	0.056	0.044	0.432	0.421	0.062	0.008	0.522	0.498	0.057
MVMR-Egger	0.018	0.235	0.228	0.059	0.038	0.401	0.392	0.056	0.081	0.521	0.511	0.064	0.056	0.628	0.604	0.066
MVMR-PRESSO	0.003	0.072	0.065	0.066	0.008	0.181	0.134	0.094	0.023	0.309	0.221	0.117	0.026	0.432	0.304	0.125
MVMR-Robust	0.003	0.053	0.051	0.069	0.004	0.077	0.078	0.058	0.010	0.213	0.237	0.021	0.019	0.468	0.466	0.050
MVMR-Median	0.003	0.066	0.071	0.038	0.006	0.090	0.088	0.055	0.008	0.135	0.116	0.080	0.011	0.261	0.168	0.175
MVMR-Lasso	0.003	0.054	0.051	0.067	0.005	0.072	0.061	0.097	0.006	0.110	0.076	0.170	0.009	0.231	0.102	0.345

**Table 3:** Point estimate, standard error (SE) and confidence interval (CI Lower, CI Upper) of the log odds ratio of Alzheimer’s disease due to a unit increase in intelligence, years of education and household income, from univariable Mendelian randomization (MR-IVW) and each multivariable method

Risk factor	F statistic	Method	Estimate	SE	CI lower	CI upper
Intelligence	31.398	MR-IVW	−0.420	0.078	−0.573	−0.267
	2.364	MVMR-IVW	−0.469	0.202	−0.864	−0.074
		MVMR-Egger	−0.073	0.332	−0.723	0.578
		MVMR-PRESSO	−0.469	0.202	−0.866	−0.072
		MVMR-Robust	−0.544	0.195	−0.927	−0.161
		MVMR-Median	−0.573	0.241	−1.045	−0.100
		MVMR-Lasso	−0.587	0.178	−0.936	−0.238
		MVMR-Q(het)	−0.095		−0.559	0.496
Years of education	21.012	MR-IVW	−0.591	0.120	−0.827	−0.355
	1.570	MVMR-IVW	−0.244	0.344	−0.919	0.430
		MVMR-Egger	−0.035	0.371	−0.761	0.691
		MVMR-PRESSO	−0.244	0.344	−0.923	0.434
		MVMR-Robust	−0.017	0.310	−0.624	0.590
		MVMR-Median	−0.134	0.384	−0.887	0.620
		MVMR-Lasso	0.179	0.301	−0.411	0.769
		MVMR-Q(het)	−0.439		−1.498	0.401
Household income	10.479	MR-IVW	−0.603	0.148	−0.894	−0.313
	1.565	MVMR-IVW	0.416	0.340	−0.250	1.082
		MVMR-Egger	0.400	0.339	−0.265	1.064
		MVMR-PRESSO	0.416	0.340	−0.254	1.086
		MVMR-Robust	0.263	0.341	−0.404	0.931
		MVMR-Median	0.368	0.381	−0.378	1.114
		MVMR-Lasso	0.097	0.298	−0.488	0.681
		MVMR-Q(het)	0.107		−0.730	0.965

*Note*: F statistics are unconditional (reported next to MR-IVW) and conditional (reported next to MVMR-IVW).

## Data Availability

The data used to support this study are available in the PhenoScanner database (http://www.phenoscanner.medschl.cam.ac.uk/)^44,45^ and the UK Biobank (see http://www.nealelab.is/uk-biobank/ and Davies etal^29^). Rcode for reproducing the simulation data is available at https://github.com/aj-grant/robust-mvmr.
