# Supplementary material for: Pleiotropy robust methods for multivariable Mendelian randomization
Source: Stat Med. Author manuscript; Available in PMC 2022 Jan 5. (PMC7612169; doi:10.1002/sim.9156)
Supplement: Supplementary File [file EMS140629-supplement-Supplementary_File.pdf]

Supplementary material to: Pleiotropy robust methods for multivariable Mendelian randomization

Andrew J. Grant*[1] and Stephen Burgess[1,2]

[1]MRC Biostatistics Unit, University of Cambridge, Cambridge, UK
[2]Cardiovascular Epidemiology Unit, University of Cambridge, Cambridge, UK

S.1 Algorithm for performing the regularization approach

Let $\hat{\boldsymbol{\beta}}_{\boldsymbol{X}}$ be the $p \times K$ matrix with $(j,\, k)^{\text{th}}$ element $\hat{\beta}_{Xjk}$, $\hat{\boldsymbol{\beta}}_{\boldsymbol{Y}}$ be the vector of length p with j^{th} element $\hat{\beta}_{Yj}$, $\boldsymbol{S}$ be the $p \times p$ diagonal matrix with $(j,\, j)^{\text{th}}$ element σ_{Yj}^{-2} and $\boldsymbol{\theta_0}$ be the vector of length p with j^{th} element θ_{0j}. We denote by $\boldsymbol{P}_{\hat{\boldsymbol{\beta}}_{\boldsymbol{X}}} = \boldsymbol{S}^{1/2}\hat{\boldsymbol{\beta}}_{\boldsymbol{X}}\left(\hat{\boldsymbol{\beta}}'_{\boldsymbol{X}}\boldsymbol{S}\hat{\boldsymbol{\beta}}_{\boldsymbol{X}}\right)^{-1}\hat{\boldsymbol{\beta}}'_{\boldsymbol{X}}\boldsymbol{S}^{1/2}$ the projection onto the column space of $\boldsymbol{S}^{1/2}\hat{\boldsymbol{\beta}}_{\boldsymbol{X}}$ and $\boldsymbol{I_p}$ the identity matrix of dimension p. We can solve (6), for a given value of λ, using the following procedure.

1. Let
$$\hat{\boldsymbol{\theta}}_{\boldsymbol{0\lambda}} = \arg\min_{\theta_0} \left\| \left(I_p - \boldsymbol{P}_{\hat{\boldsymbol{\beta}}_{\boldsymbol{X}}}\right)\boldsymbol{S}^{1/2}\left(\hat{\boldsymbol{\beta}}_{\boldsymbol{Y}} - \boldsymbol{\theta_0}\right)\right\|^2 + \lambda\sum_{j=1}^{p}|\theta_{0j}|\,,$$
where $\|\cdot\|$ denotes the ℓ_2 norm.

2. let
$$\hat{\boldsymbol{\theta}}_{\boldsymbol{\lambda}} = \left(\hat{\boldsymbol{\beta}}'_{\boldsymbol{X}}\boldsymbol{S}\hat{\boldsymbol{\beta}}_{\boldsymbol{X}}\right)^{-1}\left(\hat{\boldsymbol{\beta}}'_{\boldsymbol{X}}\boldsymbol{S}\hat{\boldsymbol{\beta}}_{\boldsymbol{Y}}\right).$$

The kth element of $\hat{\boldsymbol{\theta}}_{\boldsymbol{\lambda}}$ is the estimate of θ_k for given λ. Note that Step 1 is now a standard lasso, with responses $\left(I_p - \boldsymbol{P}_{\hat{\boldsymbol{\beta}}_{\boldsymbol{X}}}\right)\boldsymbol{S}^{1/2}\hat{\boldsymbol{\beta}}_{\boldsymbol{Y}}$ and design matrix $\left(I_p - \boldsymbol{P}_{\hat{\boldsymbol{\beta}}_{\boldsymbol{X}}}\right)\boldsymbol{S}^{1/2}$, and can be computed with standard software.

S.2 Supplementary simulation results

In this section we present the results of the supplementary simulation studies described in Section 4. Tables S1–S2 and Figure S3 show the results for the case where $p = 20$ and $\beta_{Xjk} \sim \text{Uniform}\,(0, 0.22)$.

*Corresponding author. Email address: andrew.grant@mrc-bsu.cam.ac.uk

Tables S3–S4 and Figure S4 show the results for the case where the risk factors are correlated with $\text{cor}(v_{Xik}, v_{Xil}) = 0.5$ for all $k \neq l$. Tables S5–S6 and Figure S5 show the results for the case where the genetic variant-trait associations were all estimated from the same sample (one sample Mendelian randomization). Table S7 shows a comparison of coverage and confidence interval width when performing MVMR-Median with three alternative methods for computing confidence intervals: the parametric bootstrap; a nonparametric bootstrap; and a rank inversion technique. Figure S6 illustrates a scenario where there are two risk factors and one is a mediator of the effect of the other on the outcome. Tables S8 – S9 show the results of applying the various methods to this mediation scenario for the different levels and types of pleiotropy.

S.2.1 Examining the performance of MVMR-Lasso in selecting valid and invalid instruments

We evaluate the ability for MVMR-Lasso to select which genetic variants are valid and which are invalid instruments using the following four metrics:

(a) The proportion of instruments selected as invalid which were truly invalid;

(b) The proportion of instruments selected as valid which were truly valid;

(c) The proportion of truly invalid instruments which were selected as invalid;

(d) The proportion of truly valid instruments which were estimated as valid.

The means of each of these metric for each scenario and level of pleiotropy considered in the primary simulations are shown in Table S10.

Metric (a) was close to 1 in all scenarios, suggesting that any instrument MVMR-Lasso selected as invalid is very likely to be truly invalid. Metric (d) was also close to 1 in all scenarios, suggesting that truly valid instruments are very likely to be selected as valid. Metric (b) was close to 1 in the lower pleiotropy scenarios (10% and 30%) but lower in the higher pleiotropy scenarios (50% and 70%). This suggests that, in the higher pleiotropy scenarios, some instruments which are invalid may be selected as valid, and remain in the post-lasso estimator. However, it is important to note that the pleiotropic effects in these simulations were drawn from distributions which include zero. Thus, by chance, some truly invalid instruments will have close to zero pleiotropic effect, and thus the method may be correct in some cases to consider the instrument as valid. Similarly, metric (c) will be affected by the fact that some pleiotropic effects in the simulations will be, by chance, close to zero. This metric was around 0.7–0.8 across all scenarios, however it is possible that the invalid instruments which were not selected as invalid had pleiotropic effects close to zero.

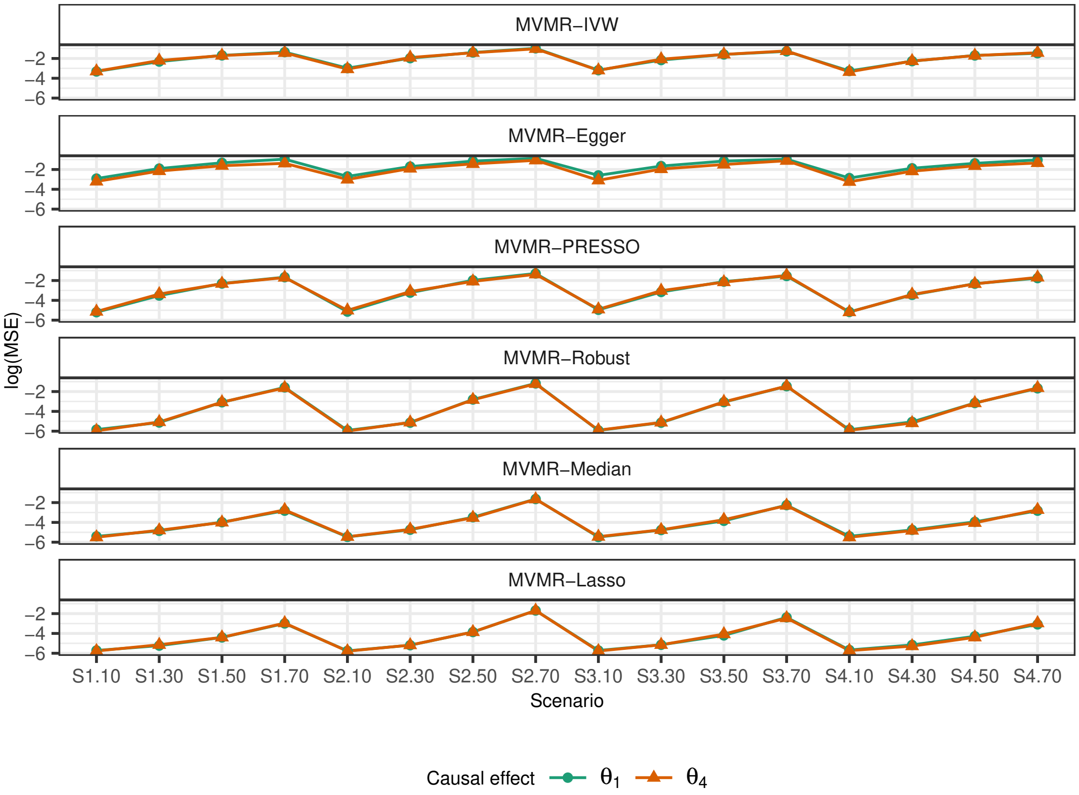

Figure S1: Logarithm of the mean squared errors for each scenario (S1, S2, S3 and S4) and proportion of invalid genetic variants (10, 30, 50 or 70%), for the causal effect estimates for the first risk factor (θ_1) and the fourth risk factor (θ_4).

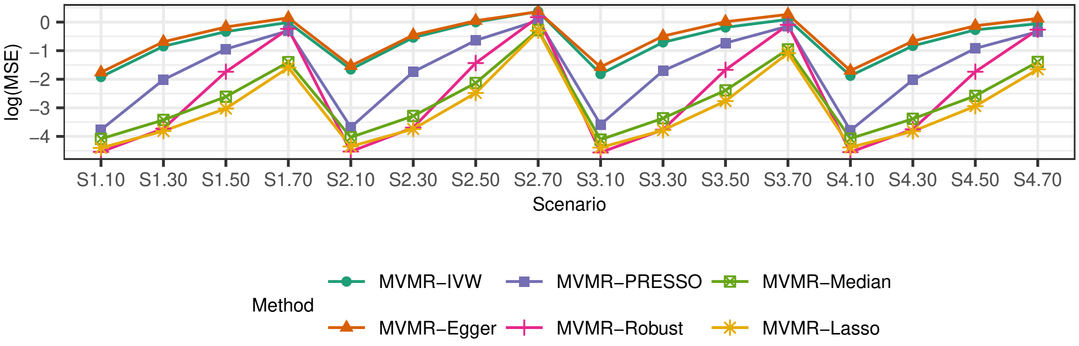

Figure S2: Logarithm of the mean squared errors for each scenario (S1, S2, S3 and S4) and proportion of invalid genetic variants (10, 30, 50 or 70%), for estimates of the full θ vector.

Table S1: Mean and standard deviation (SD) of estimates, mean standard error (SE) and power when $p = 20$ and $\theta_1 = 0.2$.

	10% invalid				30% invalid				50% invalid			
Method	Mean	SD	SE	Power	Mean	SD	SE	Power	Mean	SD	SE	Power
Scenario 1: Balanced pleiotropy, InSIDE met												
MVMR-IVW	0.198	0.224	0.203	0.280	0.207	0.398	0.363	0.129	0.188	0.491	0.474	0.084
MVMR-Egger	0.206	0.272	0.241	0.241	0.208	0.467	0.436	0.117	0.183	0.607	0.569	0.086
MVMR-PRESSO	0.200	0.132	0.103	0.602	0.196	0.319	0.247	0.225	0.195	0.453	0.369	0.148
MVMR-Robust	0.201	0.061	0.067	0.831	0.197	0.126	0.121	0.543	0.206	0.382	0.406	0.172
MVMR-Median	0.201	0.077	0.098	0.578	0.197	0.148	0.124	0.402	0.199	0.313	0.169	0.365
MVMR-Lasso	0.201	0.062	0.072	0.832	0.197	0.119	0.083	0.688	0.207	0.295	0.112	0.589
Scenario 2: Directional pleiotropy, InSIDE met												
MVMR-IVW	0.217	0.243	0.221	0.271	0.282	0.439	0.400	0.143	0.283	0.535	0.504	0.111
MVMR-Egger	0.197	0.290	0.264	0.214	0.228	0.523	0.479	0.103	0.200	0.642	0.603	0.095
MVMR-PRESSO	0.205	0.141	0.102	0.619	0.248	0.355	0.273	0.212	0.269	0.487	0.398	0.153
MVMR-Robust	0.197	0.060	0.065	0.831	0.208	0.112	0.121	0.574	0.242	0.442	0.446	0.144
MVMR-Median	0.200	0.078	0.099	0.558	0.216	0.151	0.129	0.430	0.235	0.392	0.191	0.373
MVMR-Lasso	0.198	0.060	0.073	0.818	0.207	0.124	0.085	0.700	0.230	0.386	0.123	0.544
Scenario 3: Directional pleiotropy, InSIDE violated												
MVMR-IVW	0.200	0.238	0.201	0.293	0.234	0.391	0.373	0.125	0.260	0.497	0.483	0.100
MVMR-Egger	0.209	0.281	0.240	0.244	0.248	0.480	0.445	0.115	0.247	0.588	0.571	0.089
MVMR-PRESSO	0.204	0.158	0.107	0.596	0.227	0.319	0.267	0.201	0.252	0.467	0.388	0.148
MVMR-Robust	0.203	0.065	0.065	0.834	0.195	0.118	0.121	0.552	0.244	0.407	0.399	0.172
MVMR-Median	0.205	0.081	0.099	0.587	0.204	0.143	0.125	0.418	0.240	0.348	0.180	0.362
MVMR-Lasso	0.203	0.064	0.074	0.814	0.199	0.114	0.084	0.689	0.227	0.327	0.118	0.534

Table S2: Mean and standard deviation (SD) of estimates, mean standard error (SE) and type I error rate when $p = 20$ and $\theta_1 = 0$.

	10% invalid				30% invalid				50% invalid			
Method	Mean	SD	SE	Type I	Mean	SD	SE	Type I	Mean	SD	SE	Type I
Scenario 1: Balanced pleiotropy, InSIDE met												
MVMR-IVW	0.014	0.236	0.205	0.068	-0.006	0.403	0.366	0.077	0.017	0.482	0.468	0.060
MVMR-Egger	0.013	0.280	0.245	0.053	-0.004	0.478	0.435	0.078	0.020	0.582	0.563	0.069
MVMR-PRESSO	0.006	0.134	0.099	0.073	-0.001	0.327	0.246	0.090	0.015	0.439	0.359	0.087
MVMR-Robust	0.001	0.061	0.060	0.094	0.003	0.117	0.120	0.086	-0.002	0.391	0.396	0.084
MVMR-Median	-0.001	0.076	0.084	0.025	0.003	0.138	0.108	0.061	0.007	0.324	0.155	0.196
MVMR-Lasso	0.001	0.062	0.062	0.050	-0.002	0.110	0.075	0.104	0.007	0.302	0.103	0.281
Scenario 2: Directional pleiotropy, InSIDE met												
MVMR-IVW	0.018	0.242	0.219	0.062	0.075	0.438	0.401	0.086	0.091	0.563	0.507	0.094
MVMR-Egger	0.001	0.286	0.263	0.056	0.020	0.503	0.478	0.072	-0.007	0.649	0.606	0.075
MVMR-PRESSO	0.006	0.125	0.098	0.089	0.060	0.345	0.267	0.094	0.058	0.511	0.403	0.115
MVMR-Robust	0.003	0.061	0.060	0.096	0.006	0.112	0.119	0.093	0.045	0.437	0.462	0.087
MVMR-Median	0.006	0.077	0.084	0.037	0.014	0.148	0.112	0.077	0.033	0.400	0.177	0.252
MVMR-Lasso	0.003	0.063	0.062	0.054	0.007	0.116	0.076	0.108	0.030	0.386	0.112	0.360
Scenario 3: Directional pleiotropy, InSIDE violated												
MVMR-IVW	0.017	0.235	0.212	0.070	0.040	0.394	0.370	0.073	0.072	0.533	0.487	0.078
MVMR-Egger	0.033	0.296	0.253	0.075	0.055	0.468	0.443	0.074	0.057	0.627	0.580	0.075
MVMR-PRESSO	0.011	0.130	0.103	0.082	0.024	0.323	0.258	0.093	0.067	0.498	0.392	0.100
MVMR-Robust	0.001	0.062	0.060	0.111	0.006	0.118	0.120	0.098	0.039	0.431	0.432	0.093
MVMR-Median	0.002	0.077	0.085	0.019	0.007	0.148	0.110	0.077	0.028	0.364	0.165	0.226
MVMR-Lasso	0.001	0.063	0.064	0.051	0.007	0.120	0.076	0.121	0.016	0.331	0.106	0.315

Table S3: Mean and standard deviation (SD) of estimates, mean standard error (SE) and power when the v_{Xik}'s are correlated and $\theta_1 = 0.2$.

	10% invalid				30% invalid				50% invalid			
Method	Mean	SD	SE	Power	Mean	SD	SE	Power	Mean	SD	SE	Power
Scenario 1: Balanced pleiotropy, InSIDE met												
MVMR-IVW	0.200	0.201	0.191	0.208	0.200	0.320	0.331	0.090	0.205	0.440	0.427	0.079
MVMR-Egger	0.204	0.232	0.228	0.175	0.217	0.391	0.394	0.092	0.220	0.527	0.508	0.072
MVMR-PRESSO	0.203	0.081	0.071	0.778	0.197	0.184	0.151	0.336	0.189	0.325	0.233	0.208
MVMR-Robust	0.206	0.055	0.060	0.936	0.202	0.080	0.081	0.708	0.193	0.224	0.241	0.135
MVMR-Median	0.205	0.068	0.088	0.681	0.203	0.088	0.106	0.488	0.200	0.139	0.135	0.335
MVMR-Lasso	0.206	0.061	0.063	0.908	0.204	0.079	0.073	0.793	0.203	0.115	0.088	0.607
Scenario 2: Directional pleiotropy, InSIDE met												
MVMR-IVW	0.255	0.220	0.213	0.249	0.335	0.366	0.360	0.159	0.411	0.460	0.457	0.146
MVMR-Egger	0.237	0.263	0.254	0.173	0.260	0.438	0.429	0.105	0.270	0.562	0.543	0.086
MVMR-PRESSO	0.209	0.079	0.073	0.811	0.242	0.200	0.167	0.369	0.343	0.358	0.268	0.309
MVMR-Robust	0.202	0.053	0.060	0.936	0.200	0.076	0.082	0.701	0.257	0.245	0.273	0.140
MVMR-Median	0.205	0.066	0.089	0.680	0.214	0.098	0.111	0.514	0.246	0.170	0.151	0.387
MVMR-Lasso	0.204	0.059	0.064	0.905	0.204	0.078	0.076	0.772	0.225	0.144	0.095	0.625
Scenario 3: Directional pleiotropy, InSIDE violated												
MVMR-IVW	0.240	0.207	0.197	0.256	0.295	0.345	0.340	0.154	0.314	0.450	0.433	0.114
MVMR-Egger	0.273	0.261	0.233	0.257	0.351	0.425	0.395	0.176	0.369	0.530	0.499	0.127
MVMR-PRESSO	0.208	0.086	0.077	0.774	0.253	0.219	0.174	0.361	0.285	0.357	0.271	0.260
MVMR-Robust	0.203	0.053	0.060	0.941	0.208	0.082	0.082	0.732	0.235	0.217	0.248	0.142
MVMR-Median	0.205	0.067	0.089	0.688	0.219	0.099	0.109	0.523	0.226	0.148	0.141	0.375
MVMR-Lasso	0.204	0.060	0.064	0.913	0.207	0.082	0.075	0.773	0.217	0.123	0.091	0.628

Table S4: Mean and standard deviation (SD) of estimates, mean standard error (SE) and type I error rate when the v_{Xik}'s are correlated and $\theta_1 = 0$.

	10% invalid				30% invalid				50% invalid			
Method	Mean	SD	SE	Type I	Mean	SD	SE	Type I	Mean	SD	SE	Type I
Scenario 1: Balanced pleiotropy, InSIDE met												
MVMR-IVW	0.007	0.202	0.193	0.059	0.009	0.332	0.332	0.043	0.007	0.440	0.425	0.056
MVMR-Egger	0.015	0.235	0.230	0.058	0.013	0.399	0.396	0.054	0.005	0.523	0.507	0.059
MVMR-PRESSO	0.004	0.076	0.067	0.069	0.003	0.183	0.142	0.092	-0.002	0.311	0.225	0.112
MVMR-Robust	0.003	0.054	0.053	0.056	0.001	0.082	0.081	0.042	-0.005	0.220	0.241	0.026
MVMR-Median	0.004	0.070	0.074	0.040	0.005	0.095	0.091	0.053	-0.002	0.142	0.118	0.088
MVMR-Lasso	0.003	0.055	0.053	0.060	0.003	0.076	0.063	0.092	-0.002	0.114	0.077	0.162
Scenario 2: Directional pleiotropy, InSIDE met												
MVMR-IVW	0.046	0.221	0.212	0.064	0.119	0.377	0.362	0.074	0.242	0.472	0.454	0.101
MVMR-Egger	0.028	0.264	0.253	0.056	0.044	0.448	0.431	0.066	0.113	0.565	0.541	0.066
MVMR-PRESSO	0.006	0.079	0.070	0.052	0.042	0.206	0.163	0.089	0.137	0.352	0.259	0.139
MVMR-Robust	0.004	0.053	0.053	0.052	0.003	0.083	0.082	0.050	0.065	0.244	0.269	0.016
MVMR-Median	0.006	0.068	0.074	0.024	0.018	0.098	0.095	0.062	0.053	0.177	0.132	0.137
MVMR-Lasso	0.004	0.055	0.053	0.058	0.004	0.077	0.065	0.108	0.032	0.148	0.084	0.241
Scenario 3: Directional pleiotropy, InSIDE violated												
MVMR-IVW	0.047	0.203	0.196	0.055	0.087	0.350	0.340	0.074	0.132	0.451	0.432	0.070
MVMR-Egger	0.082	0.266	0.232	0.089	0.152	0.429	0.396	0.091	0.177	0.529	0.499	0.079
MVMR-PRESSO	0.014	0.087	0.073	0.070	0.043	0.209	0.166	0.092	0.079	0.346	0.262	0.108
MVMR-Robust	0.007	0.055	0.053	0.052	0.012	0.083	0.082	0.053	0.029	0.225	0.248	0.015
MVMR-Median	0.010	0.067	0.073	0.032	0.019	0.099	0.093	0.065	0.026	0.150	0.123	0.100
MVMR-Lasso	0.007	0.056	0.052	0.057	0.012	0.078	0.064	0.106	0.015	0.126	0.080	0.196

Table S5: Mean and standard deviation (SD) of estimates, mean standard error (SE) and power when the genetic variant-risk factor and genetic variant-outcome associations are estimated in the same sample and $\theta_1 = 0.2$.

	10% invalid				30% invalid				50% invalid			
Method	Mean	SD	SE	Power	Mean	SD	SE	Power	Mean	SD	SE	Power
Scenario 1: Balanced pleiotropy, InSIDE met												
MVMR-IVW	0.209	0.194	0.185	0.231	0.203	0.328	0.320	0.112	0.213	0.403	0.413	0.078
MVMR-Egger	0.226	0.237	0.225	0.210	0.215	0.403	0.390	0.105	0.234	0.489	0.502	0.071
MVMR-PRESSO	0.202	0.074	0.068	0.831	0.197	0.184	0.138	0.392	0.198	0.306	0.220	0.248
MVMR-Robust	0.203	0.053	0.055	0.960	0.199	0.078	0.078	0.726	0.191	0.217	0.230	0.159
MVMR-Median	0.203	0.066	0.082	0.752	0.198	0.090	0.100	0.528	0.200	0.132	0.126	0.374
MVMR-Lasso	0.203	0.055	0.058	0.945	0.199	0.074	0.068	0.801	0.197	0.113	0.082	0.636
Scenario 2: Directional pleiotropy, InSIDE met												
MVMR-IVW	0.263	0.217	0.207	0.282	0.346	0.357	0.351	0.181	0.438	0.440	0.441	0.171
MVMR-Egger	0.248	0.251	0.251	0.186	0.272	0.424	0.426	0.096	0.305	0.540	0.534	0.102
MVMR-PRESSO	0.206	0.077	0.069	0.809	0.257	0.206	0.159	0.442	0.337	0.337	0.256	0.337
MVMR-Robust	0.202	0.053	0.056	0.950	0.206	0.079	0.079	0.745	0.252	0.231	0.257	0.146
MVMR-Median	0.206	0.067	0.083	0.737	0.222	0.098	0.105	0.569	0.247	0.163	0.142	0.435
MVMR-Lasso	0.203	0.057	0.059	0.923	0.207	0.076	0.071	0.819	0.227	0.136	0.089	0.672
Scenario 3: Directional pleiotropy, InSIDE violated												
MVMR-IVW	0.235	0.201	0.192	0.262	0.282	0.329	0.329	0.138	0.324	0.432	0.422	0.119
MVMR-Egger	0.275	0.259	0.231	0.256	0.350	0.417	0.390	0.175	0.392	0.518	0.494	0.142
MVMR-PRESSO	0.209	0.083	0.074	0.799	0.241	0.202	0.162	0.403	0.285	0.350	0.254	0.284
MVMR-Robust	0.203	0.052	0.055	0.961	0.208	0.076	0.079	0.766	0.233	0.220	0.250	0.132
MVMR-Median	0.205	0.065	0.083	0.745	0.219	0.089	0.102	0.592	0.231	0.152	0.135	0.420
MVMR-Lasso	0.205	0.057	0.059	0.939	0.210	0.075	0.070	0.836	0.222	0.131	0.086	0.661

Table S6: Mean and standard deviation (SD) of estimates, mean standard error (SE) and type I error rate when the genetic variant-risk factor and genetic variant-outcome associations are estimated in the same sample and $\theta_1 = 0$.

	10% invalid				30% invalid				50% invalid			
Method	Mean	SD	SE	Type I	Mean	SD	SE	Type I	Mean	SD	SE	Type I
Scenario 1: Balanced pleiotropy, InSIDE met												
MVMR-IVW	0.000	0.200	0.188	0.066	0.001	0.345	0.323	0.057	-0.007	0.427	0.414	0.059
MVMR-Egger	0.018	0.240	0.229	0.058	-0.006	0.410	0.392	0.054	0.009	0.513	0.503	0.049
MVMR-PRESSO	-0.003	0.072	0.065	0.053	0.002	0.182	0.136	0.079	-0.001	0.301	0.216	0.105
MVMR-Robust	0.003	0.050	0.052	0.047	0.002	0.075	0.078	0.051	0.006	0.210	0.235	0.018
MVMR-Median	0.003	0.066	0.072	0.031	0.003	0.094	0.089	0.052	-0.001	0.146	0.115	0.117
MVMR-Lasso	0.003	0.051	0.051	0.051	0.001	0.073	0.061	0.092	0.002	0.116	0.076	0.183
Scenario 2: Directional pleiotropy, InSIDE met												
MVMR-IVW	0.053	0.215	0.206	0.067	0.140	0.366	0.350	0.075	0.248	0.433	0.441	0.079
MVMR-Egger	0.037	0.258	0.250	0.051	0.087	0.438	0.426	0.054	0.117	0.542	0.535	0.066
MVMR-PRESSO	0.007	0.073	0.066	0.063	0.045	0.200	0.152	0.090	0.131	0.326	0.244	0.147
MVMR-Robust	0.003	0.052	0.052	0.061	0.010	0.081	0.080	0.048	0.058	0.234	0.261	0.027
MVMR-Median	0.006	0.067	0.072	0.035	0.020	0.097	0.092	0.061	0.052	0.165	0.128	0.121
MVMR-Lasso	0.003	0.053	0.052	0.056	0.011	0.076	0.063	0.108	0.033	0.139	0.081	0.242
Scenario 3: Directional pleiotropy, InSIDE violated												
MVMR-IVW	0.028	0.208	0.192	0.059	0.108	0.343	0.328	0.069	0.119	0.420	0.418	0.063
MVMR-Egger	0.067	0.270	0.230	0.097	0.178	0.426	0.390	0.097	0.179	0.501	0.490	0.071
MVMR-PRESSO	0.007	0.078	0.071	0.061	0.050	0.203	0.156	0.092	0.077	0.324	0.248	0.119
MVMR-Robust	0.002	0.052	0.052	0.059	0.003	0.076	0.079	0.049	0.023	0.213	0.242	0.031
MVMR-Median	0.002	0.065	0.072	0.021	0.016	0.092	0.090	0.056	0.030	0.147	0.119	0.110
MVMR-Lasso	0.002	0.053	0.051	0.067	0.006	0.071	0.062	0.092	0.016	0.118	0.077	0.191

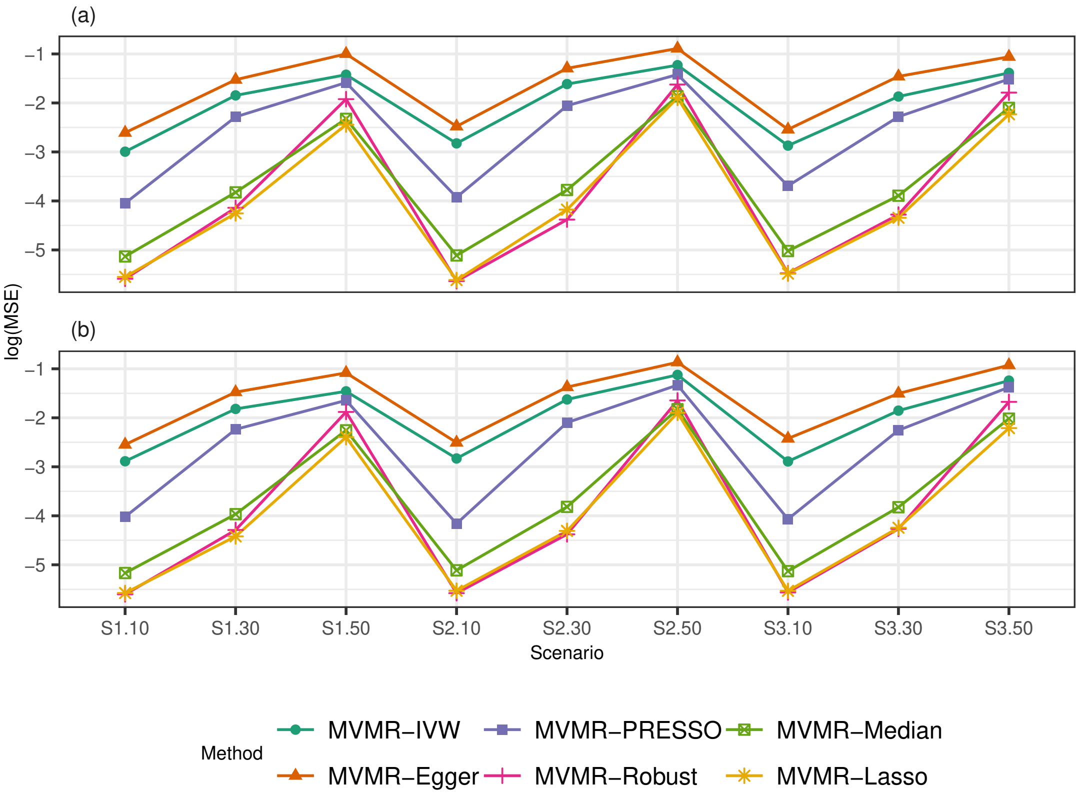

Figure S3: Logarithm of the mean squared errors for each scenario (S1, S2 and S3) and proportion of invalid genetic variants (10, 30 or 50%), when $p = 20$ and (a) $\theta_1 = 0.2$ and (b) $\theta_1 = 0$.

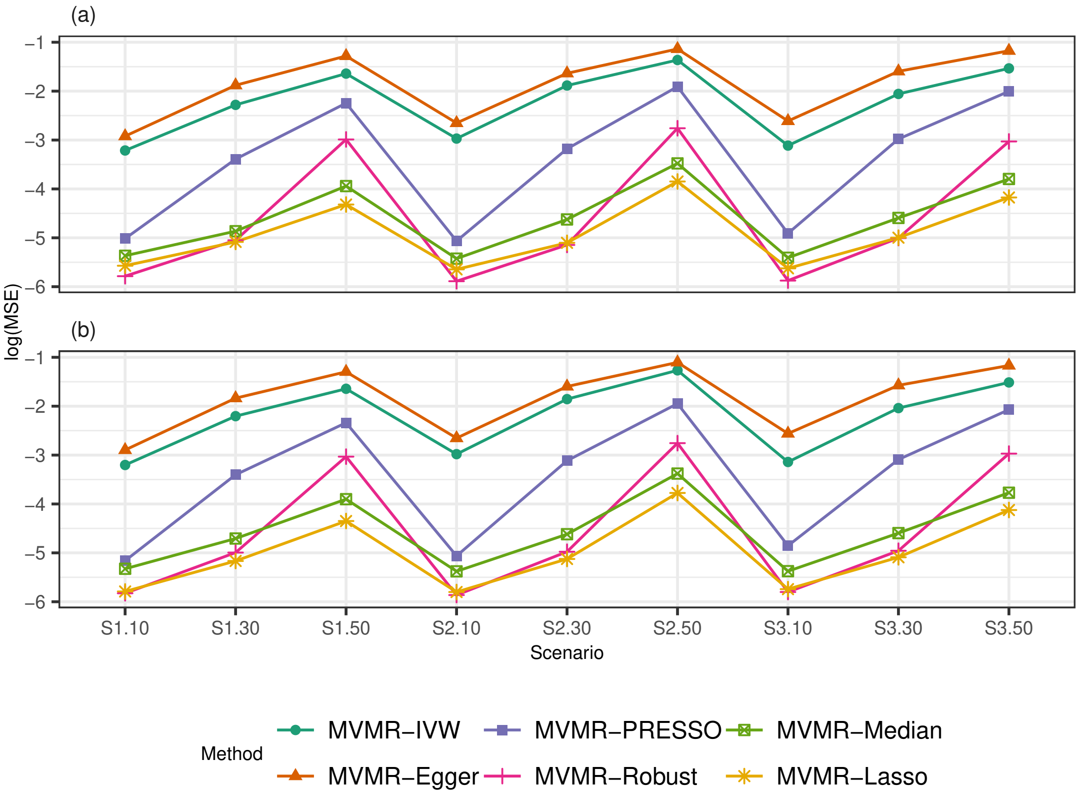

Figure S4: Logarithm of the mean squared errors for each scenario (S1, S2 and S3) and proportion of invalid genetic variants (10, 30 or 50%), when the ε_{Wij}'s are correlated and (a) $\theta_1 = 0.2$ and (b) $\theta_1 = 0$.

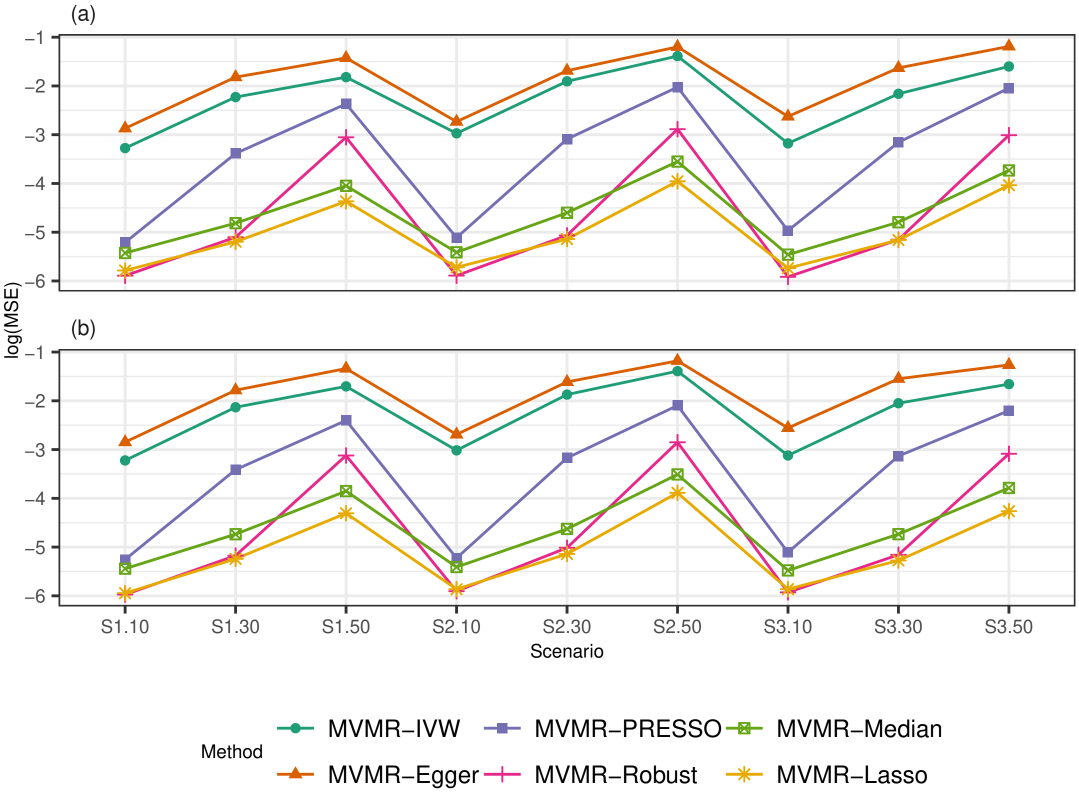

Figure S5: Logarithm of the mean squared errors for each scenario (S1, S2 and S3) and proportion of invalid genetic variants (10, 30 or 50%), when the genetic variant-risk factor and genetic variant-outcome associations are estimated in the same sample and (a) $\theta_1 = 0.2$ and (b) $\theta_1 = 0$.

Table S7: Mean coverage and confidence interval (CI) width for MVMR-Median using three alternative methods for computing confidence intervals.

		Parametric bootstrap		Nonarametric bootstrap		Rank inversion	
θ	Invalid	Coverage	CI width	Coverage	CI width	Coverage	CI width
		Scenario 1: Balanced pleiotropy, InSIDE met					
0.2	10%	0.983	0.161	0.923	0.232	0.879	0.208
	30%	0.974	0.196	0.947	0.333	0.889	0.291
	50%	0.948	0.250	0.944	0.572	0.884	0.464
		Scenario 2: Directional pleiotropy, InSIDE met					
	10%	0.987	0.164	0.944	0.235	0.899	0.210
	30%	0.975	0.206	0.936	0.358	0.870	0.312
	50%	0.906	0.279	0.916	0.729	0.849	0.561
		Scenario 3: Directional pleiotropy, InSIDE violated					
	10%	0.985	0.163	0.939	0.234	0.889	0.208
	30%	0.973	0.201	0.939	0.348	0.884	0.301
	50%	0.938	0.260	0.947	0.622	0.888	0.495
		Scenario 1: Balanced pleiotropy, InSIDE met					
0	10%	0.962	0.140	0.934	0.232	0.856	0.206
	30%	0.946	0.173	0.938	0.333	0.883	0.290
	50%	0.907	0.224	0.929	0.562	0.872	0.453
		Scenario 2: Directional pleiotropy, InSIDE met					
	10%	0.974	0.142	0.949	0.239	0.888	0.214
	30%	0.940	0.181	0.930	0.358	0.873	0.310
	50%	0.874	0.252	0.923	0.740	0.859	0.570
		Scenario 3: Directional pleiotropy, InSIDE violated					
	10%	0.970	0.140	0.939	0.234	0.872	0.209
	30%	0.934	0.176	0.927	0.347	0.873	0.301
	50%	0.896	0.235	0.943	0.630	0.882	0.501

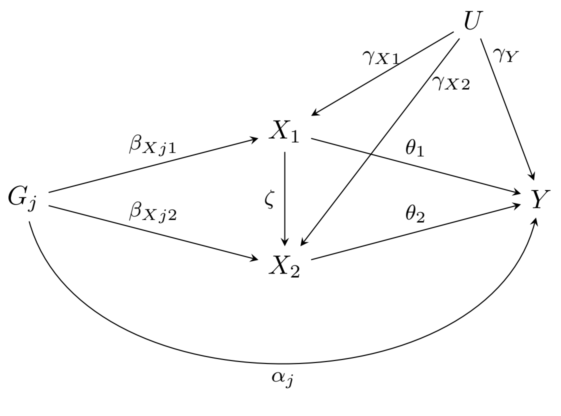

Figure S6: Directed acyclic graph illustrating the relationship between the jth genetic variant (G_j), the risk factors (X_1, X_2), confounders (U) and the outcome (Y) in the simulated scenarios with mediation. In these scenarios, $K = 2$, $\theta_1 = 0.2$ or 0, $\theta_2 = 0.2$, $\zeta = 1$ and all other parameters are the same as in the primary simulation study.

Table S8: Mean and standard deviation (SD) of estimates, mean standard error (SE) and power when there is mediation and $\theta_1 = 0.2$.

	10% invalid				30% invalid				50% invalid			
Method	Mean	SD	SE	Power	Mean	SD	SE	Power	Mean	SD	SE	Power
Scenario 1: Balanced pleiotropy, InSIDE met												
MVMR-IVW	0.202	0.318	0.295	0.120	0.181	0.527	0.509	0.076	0.184	0.697	0.660	0.077
MVMR-Egger	0.209	0.330	0.307	0.117	0.190	0.547	0.529	0.069	0.189	0.713	0.685	0.068
MVMR-PRESSO	0.202	0.318	0.295	0.117	0.181	0.527	0.509	0.072	0.184	0.697	0.660	0.076
MVMR-Robust	0.196	0.084	0.087	0.640	0.195	0.123	0.120	0.403	0.177	0.333	0.346	0.089
MVMR-Median	0.197	0.103	0.116	0.398	0.198	0.144	0.144	0.292	0.187	0.222	0.191	0.198
MVMR-Lasso	0.197	0.093	0.092	0.572	0.195	0.117	0.107	0.458	0.193	0.179	0.130	0.371
Scenario 2: Directional pleiotropy, InSIDE met												
MVMR-IVW	0.190	0.363	0.331	0.119	0.194	0.552	0.555	0.061	0.207	0.705	0.704	0.068
MVMR-Egger	0.173	0.374	0.344	0.111	0.128	0.568	0.576	0.053	0.091	0.730	0.731	0.051
MVMR-PRESSO	0.190	0.363	0.331	0.114	0.194	0.552	0.555	0.058	0.207	0.705	0.704	0.059
MVMR-Robust	0.197	0.083	0.088	0.620	0.201	0.118	0.123	0.410	0.203	0.347	0.379	0.073
MVMR-Median	0.197	0.107	0.118	0.386	0.204	0.146	0.151	0.262	0.203	0.241	0.212	0.182
MVMR-Lasso	0.195	0.089	0.094	0.570	0.201	0.114	0.111	0.437	0.197	0.199	0.138	0.366
Scenario 3: Directional pleiotropy, InSIDE violated												
MVMR-IVW	0.202	0.321	0.308	0.101	0.189	0.535	0.523	0.074	0.212	0.683	0.671	0.069
MVMR-Egger	0.242	0.341	0.318	0.126	0.249	0.564	0.536	0.094	0.253	0.706	0.683	0.080
MVMR-PRESSO	0.202	0.321	0.308	0.098	0.189	0.535	0.523	0.072	0.212	0.683	0.671	0.064
MVMR-Robust	0.202	0.084	0.088	0.641	0.201	0.126	0.123	0.412	0.211	0.327	0.352	0.079
MVMR-Median	0.203	0.107	0.117	0.415	0.203	0.149	0.148	0.294	0.206	0.223	0.201	0.195
MVMR-Lasso	0.204	0.090	0.093	0.594	0.200	0.122	0.109	0.449	0.208	0.193	0.134	0.402

Table S9: Mean and standard deviation (SD) of estimates, mean standard error (SE) and type I error rate when there is mediation and $\theta_1 = 0$.

	10% invalid				30% invalid				50% invalid			
Method	Mean	SD	SE	Type I	Mean	SD	SE	Type I	Mean	SD	SE	Type I
Scenario 1: Balanced pleiotropy, InSIDE met												
MVMR-IVW	0.005	0.304	0.294	0.054	-0.019	0.519	0.514	0.052	-0.020	0.690	0.662	0.061
MVMR-Egger	0.016	0.317	0.306	0.061	-0.009	0.536	0.534	0.049	-0.002	0.711	0.688	0.062
MVMR-PRESSO	0.005	0.304	0.294	0.053	-0.019	0.519	0.514	0.050	-0.020	0.690	0.662	0.059
MVMR-Robust	0.002	0.085	0.083	0.063	-0.002	0.126	0.122	0.046	-0.017	0.301	0.336	0.022
MVMR-Median	0.003	0.105	0.109	0.049	-0.005	0.147	0.138	0.061	-0.010	0.210	0.182	0.080
MVMR-Lasso	0.002	0.088	0.086	0.061	-0.004	0.116	0.102	0.079	-0.005	0.168	0.124	0.141
Scenario 2: Directional pleiotropy, InSIDE met												
MVMR-IVW	-0.007	0.350	0.332	0.055	0.014	0.576	0.558	0.062	0.038	0.752	0.704	0.068
MVMR-Egger	-0.024	0.364	0.345	0.054	-0.052	0.595	0.579	0.061	-0.086	0.773	0.730	0.070
MVMR-PRESSO	-0.007	0.350	0.332	0.052	0.014	0.576	0.558	0.060	0.038	0.752	0.704	0.066
MVMR-Robust	0.001	0.085	0.084	0.055	-0.002	0.124	0.124	0.048	0.018	0.360	0.386	0.022
MVMR-Median	0.002	0.107	0.111	0.034	0.001	0.155	0.145	0.065	0.012	0.253	0.206	0.092
MVMR-Lasso	0.002	0.089	0.088	0.051	-0.001	0.117	0.106	0.076	0.002	0.210	0.132	0.178
Scenario 3: Directional pleiotropy, InSIDE violated												
MVMR-IVW	0.003	0.322	0.303	0.055	0.033	0.520	0.521	0.058	-0.019	0.692	0.664	0.066
MVMR-Egger	0.038	0.341	0.313	0.063	0.091	0.542	0.535	0.066	0.031	0.712	0.676	0.067
MVMR-PRESSO	0.003	0.322	0.303	0.052	0.033	0.520	0.521	0.055	-0.019	0.692	0.664	0.064
MVMR-Robust	-0.002	0.080	0.084	0.047	0.006	0.120	0.122	0.041	-0.004	0.329	0.358	0.030
MVMR-Median	-0.002	0.103	0.110	0.033	0.005	0.142	0.140	0.049	-0.005	0.226	0.194	0.085
MVMR-Lasso	-0.001	0.083	0.087	0.037	0.007	0.114	0.103	0.072	-0.005	0.187	0.128	0.165

Table S10: Metrics evaluating the ability for MVMR-Lasso to correctly select instruments as valid or invalid. The values are the mean of each metric for each scenario and level of pleiotropy.

θ	Invalid	(a)	(b)	(c)	(d)
	Scenario 1: Balanced pleiotropy, InSIDE met				
0.2	10	0.997	0.967	0.693	1.000
	30	0.990	0.909	0.766	0.996
	50	0.974	0.828	0.794	0.978
	70	0.961	0.674	0.806	0.921
	Scenario 2: Directional pleiotropy, InSIDE met				
	10	0.999	0.970	0.718	1.000
	30	0.985	0.913	0.776	0.995
	50	0.933	0.828	0.803	0.938
	70	0.868	0.610	0.811	0.699
	Scenario 3: Directional pleiotropy, InSIDE violated				
	10	0.998	0.968	0.702	1.000
	30	0.987	0.909	0.765	0.995
	50	0.961	0.824	0.792	0.965
	70	0.929	0.652	0.805	0.849
	Scenario 4: Balanced pleiotropy, InSIDE violated				
	10	0.999	0.968	0.698	1.000
	30	0.989	0.909	0.766	0.996
	50	0.974	0.826	0.792	0.978
	70	0.963	0.678	0.809	0.923
	Scenario 1: Balanced pleiotropy, InSIDE met				
0	10	0.938	0.979	0.804	0.992
	30	0.948	0.926	0.817	0.978
	50	0.950	0.847	0.825	0.954
	70	0.948	0.692	0.827	0.889
	Scenario 2: Directional pleiotropy, InSIDE met				
	10	0.934	0.980	0.821	0.991
	30	0.942	0.932	0.832	0.976
	50	0.906	0.847	0.834	0.907
	70	0.853	0.618	0.830	0.653
	Scenario 3: Directional pleiotropy, InSIDE violated				
	10	0.938	0.979	0.803	0.992
	30	0.946	0.929	0.824	0.978
	50	0.930	0.847	0.829	0.933
	70	0.914	0.671	0.829	0.809
	Scenario 4: Balanced pleiotropy, InSIDE violated				
	10	0.932	0.979	0.806	0.991
	30	0.950	0.927	0.819	0.979
	50	0.950	0.848	0.827	0.954
	70	0.948	0.696	0.831	0.888

(a) The proportion of instruments selected as invalid which were truly invalid
(b) The proportion of instruments selected as valid which were truly valid
(c) The proportion of truly invalid instruments which were selected as invalid
(d) The proportion of truly valid instruments which were estimated as valid.

S.3 Assessing the degree of multicollinearity of the genetic variant-risk factor associations

The determinant of the matrix $\hat{\boldsymbol{\beta}}_{\boldsymbol{X}}{}'\hat{\boldsymbol{\beta}}_{\boldsymbol{X}}$ will decrease as the degree of multicollinearity in the genetic variant-risk factor association estimates increases.[1] Since the inverse of this matrix is required for each of the estimators we consider, very small determinants will lead to numerical instability of the estimates. Perfect multicollinearity will lead to a zero determinant, and suggests that the full rank condition given in Section 2.1 may be violated. We therefore require that the eigenvalues of $\hat{\boldsymbol{\beta}}_{\boldsymbol{X}}{}'\hat{\boldsymbol{\beta}}_{\boldsymbol{X}}$ are sufficiently far from zero. A common way to assess this is to use the condition index, defined as $\sqrt{\lambda_{\max}/\lambda_k}$, where λ_k is the kth eigenvalue of $\hat{\boldsymbol{\beta}}_{\boldsymbol{X}}{}'\hat{\boldsymbol{\beta}}_{\boldsymbol{X}}$ and $\lambda_{\max}$ is the maximum eigenvalue. The largest condition index is the condition number, and the condition number will increase as the degree of multicollinearity increases.

Table S11 shows the eigenvalues and their condition index computed using the genetic association estimates from the applied example of Section 5. The condition number is 6.8, which is below the threshold of 30 at which the degree of multicollinearity is typically considered to be of concern.

Table S11: Eigenvalues and condition indices for the matrix of genetic association estimates from the applied example.

Eigenvalue	Condition Index
0.152	1.000
0.007	4.776
0.003	6.775

[1] Johnston, J. *Econometric Methods.* 3rd ed. Singapore: McGraw-Hill; 1984
